# Towards a Molecular Understanding of the Link between Imatinib Resistance and Kinase Conformational Dynamics

**DOI:** 10.1371/journal.pcbi.1004578

**Published:** 2015-11-25

**Authors:** Silvia Lovera, Maria Morando, Encarna Pucheta-Martinez, Jorge L. Martinez-Torrecuadrada, Giorgio Saladino, Francesco L. Gervasio

**Affiliations:** 1 Department of Chemistry, University College London, London, United Kingdom; 2 Center of Technological Development in Health, Oswaldo Cruz Foundation (Fiocruz), Rio de Janeiro, Brazil; 3 Proteomics Core Unit, Spanish National Cancer Research Centre (CNIO), Madrid, Spain; 4 Institute of Structural and Molecular Biology, University College London, London, United Kingdom; US Army Medical Research and Materiel Command, UNITED STATES

## Abstract

Due to its inhibition of the Abl kinase domain in the BCR-ABL fusion protein, imatinib is strikingly effective in the initial stage of chronic myeloid leukemia with more than 90% of the patients showing complete remission. However, as in the case of most targeted anti-cancer therapies, the emergence of drug resistance is a serious concern. Several drug-resistant mutations affecting the catalytic domain of Abl and other tyrosine kinases are now known. But, despite their importance and the adverse effect that they have on the prognosis of the cancer patients harboring them, the molecular mechanism of these mutations is still debated. Here by using long molecular dynamics simulations and large-scale free energy calculations complemented by *in vitro* mutagenesis and microcalorimetry experiments, we model the effect of several widespread drug-resistant mutations of Abl. By comparing the conformational free energy landscape of the mutants with those of the wild-type tyrosine kinases we clarify their mode of action. It involves significant and complex changes in the inactive-to-active dynamics and entropy/enthalpy balance of two functional elements: the activation-loop and the conserved DFG motif. What is more the T315I gatekeeper mutant has a significant impact on the binding mechanism itself and on the binding kinetics.

## Author Summary

Imatinib remains the most important and studied anti-cancer drug for cancer therapy in its new paradigm. Due to its inhibition of the Abl kinase domain, imatinib is strikingly effective in the initial stage of chronic myeloid leukemia with more than 90% of the patients showing a complete remission. However, the emergence of drug resistance is a serious concern. Here, we investigate the molecular mechanism of drug-resistant mutations which, despite the importance and the adverse effect on cancer patients prognosis, is still debated. Our extensive molecular simulations and free energy calculations are consistent with an allosteric effect of the single-point drug-resistance-causing mutations on the conformational dynamics. Two partially independent conformational changes play a role. Our findings might help the design of anti-cancer therapies to overcome drug resistance and be used to predict the clinical relevance of new drug-resistant mutants found by genetic screenings of tumor samples.

## Introduction

**Fig 1:**
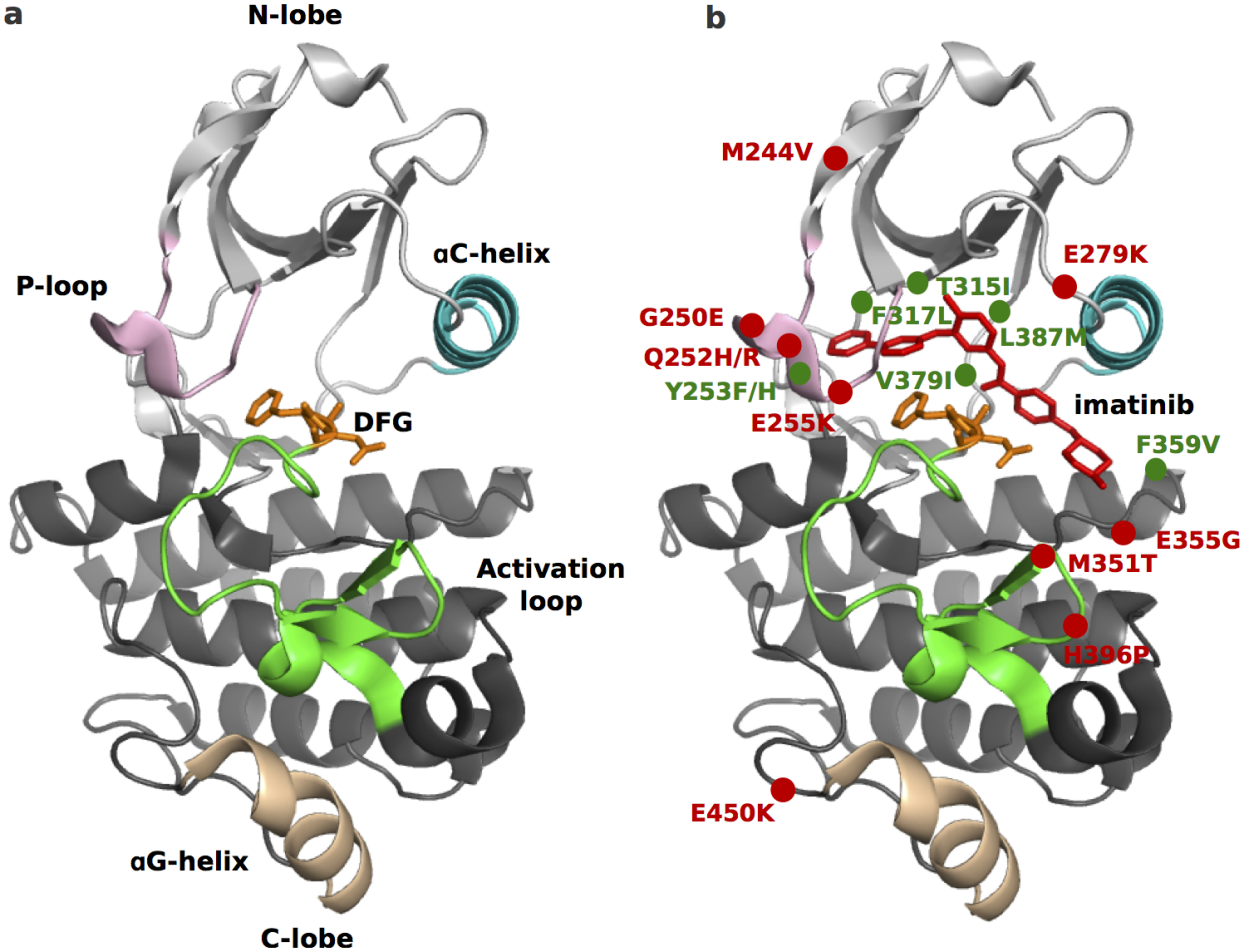
Abl structure and location of drug-resistant mutations. The main structural features, including the regions undergoing conformational changes are highlighted in different colors (a). On the right (b) imatinib binding mode and the position of drug-resistant mutants are shown. The mutants with a “known” mechanism of action are depicted in green, those for which the mechanism is still unknown in red.

The revolutionary discovery of the potent anticancer drug imatinib (Gleevec, 2001) [1] had a huge impact on cancer therapy. This drug has a striking efficacy in the early stages of chronic myeloid leukemia (CML), with 90% of patients showing remission [2, 3]. Imatinib targets the Abl tyrosine kinase (TK), constitutively active in CML due to a chromosomal translocation [4]. Unfortunately, most patients in an advanced stage of the disease suffer from relapse due to the onset of drug-resistance [5]. Even if, next-generation kinase inhibitors (KIs) are available, or in clinical trials [6], their efficacy might also be affected by drug resistance responses. Among different mechanisms, the development of resistance-inducing mutations is the most relevant in tyrosine kinases [6]. Mutations occur in highly conserved positions on the protein [7], frequently shared by several kinases [8], suggesting a conserved kinome-wide mechanism. Unfortunately, the molecular mechanism of mutation-mediated resistance are only partially understood. In the case of the widely studied “gatekeeper” mutant, found in several TKs (T315I in Abl) [9], three mechanisms have been proposed. The *direct* one involves the abrogation of a crucial hydrogen bond formed by imatinib. A second hypothesis posits that the observed shift towards the active form, which was reported in Abl and several other TK bearing the gatekeeper mutation, would allow the natural substrate ATP to outcompete the inhibitors. [10–13] Very recently, a third mechanism has been proposed for Abl T315I whereby the suppression of an induced fit effect involving the p-loop would be responsible for the decreased binding affinity of imatinib. [14] It is probable that the gate-keeper mutations have a combined effect on the binding of inhibitors, changing their binding mode and affecting at the same time the conformational changes [10, 11]. The importance of the conformational changes in the mode of action of drug-resistant mutations [15, 16] is also confirmed by the fact that many of them are far away from the binding site (Fig 1), and thus act allosterically by disfavoring the drug-binding conformation and favoring active form [8, 17–19]. The link between conformational changes and allosteric regulation in TKs is well established. For instance, in the case of Src (a close homologue of Abl) the gatekeeper mutation has been shown to allosterically affect remote regulatory motifs [20].

Indeed, TKs can exist in a dynamic equilibrium between multiple conformations [21–23], differing by the conformation of the “activation loop” (A-loop), of the conserved DFG motif and of the *α*C-helix (Fig 1). While the features of the active, catalytically-competent, state are shared among different TKs, and include an A-loop in an extended (“open”) conformation, inactive conformations can be multiple and highly diverse, all sharing an (at least partially) “closed” A-loop. Type II inhibitors, as imatinib, target a particular inactive conformation, known as “DFG-out”, where the aspartate is flipped, taking the place of the phenylalanine and pointing out of the ATP binding site [24] and opening an adjacent “allosteric” pocket. When both the DFG is in the ‘out’ conformation and the A-loop is not fully extended, the drug can enter the cavity and adopt a bridge position above the DFG, occupying both the catalytic site and the allosteric pocket [25]. The DFG-out conformation has been observed in many kinases but, despite a common binding mode, imatinib binds strongly only to some of them [26]. In contrast to the induced-fit mechanism proposed for Abl, free energy calculations performed with different and independent approaches on several TKs are consistent with a conformational selection mechanism [23, 27, 28]. Indeed, the different affinity to Abl and Src can be explained by the thermodynamic penalty to adopt the drug-binding “DFG-out” conformation [29]. Our simulations on Abl and Src also revealed a correlation between the flexibility of specific functionally-relevant structural elements and the DFG-out penalty [29].

To investigate the molecular mechanism of drug-resistant mutations, and whether or not changes in the conformational landscape, have a role in it, here we performed enhanced-sampling atomistic molecular dynamics (MD) simulations and free energy calculations on a pool of wild-type (WT) TKs for which imatinib has various strengths (IC_50_), and several Abl Imatinib-resistant mutants. In most drug resistant mutants we find extensive changes in the conformational free energy landscapes associated with two functionally-important conformational changes: the DFG-flip and the closed to open A-loop switch. The changes between the relative free energies of the active (DFG-in, extended A-loop) and inactive (DFG-out, closed A-loop) states are in the main due to entropic contributions arising from the fast (sub-*μ*s) dynamics of proximal structural elements. It is thus not surprising that we find a correlation between the sub-*μ*s flexibility of specific structural elements in the active state and drug resistance. Indeed, altering the sub-*μ*s dynamics has an effect on the binding of Imatinib, as also shown by mutagenesis and calorimetry experiments. The gatekeeper mutant T315I is a notable exception, as it affects both the binding mechanism itself and the conformation of the A-loop equilibrium. To the best of our knowledge this is the most in-depth computational study addressing the molecular roots of resistant mutants of Abl.

## Materials and Methods

### Molecular Dynamics Simulations

The kinase structures were retrieved from the Protein Data Bank (PDB entries 2G1T, 2SRC, 1PKG, 3KMM, 2WGJ and 2ITW). Missing residues were added using the software Modeller [30], according to the respective Uniprot sequences. We used the Amber99SB*-ILDN [31, 32] force field, including backbone corrections by Hummer and Best [33], with explicit TIP3P [34] water molecules. The unbiased MD simulations were carried out with the ACEMD program [35] running on GPUs. The systems were minimized with 10000 steps of conjugated gradient and equilibrated in the isothermal-isobaric (NPT) ensemble for 10 ns, using a Berendsen barostat at 1 atm. The temperature was kept at 305 K by a Langevin thermostat. A 400 ns production run was carried out for all the systems in the canonical (NVT) ensemble. The runs for Abl, Src and Kit were extended to a total length of 1 *μ*s in both the DFG-in and DFG-out conformations. The simulations of the five Abl mutants (G250E, E279K, H396P, E450K and T315I) were carried out with the same setup in both the DFG-in and DFG-out conformation, after mutating in-silico the respective residue in the Abl structure. To capture the sub-*μ*s dynamics, the RMSF of the *C*_*α*_ were averaged on 30ns non-overlapping windows after discarding the first 100ns of each run. We also compared the diffusion in the space described by the first two principal component analysis vectors (see S9 Fig).

Since the conformational changes of TKs take place on time scales longer than those accessible by standard MD simulations, here we used a combination of enhanced sampling approaches. Parallel Tempering—Well Tempered Metadynamics [36, 37] in the well tempered ensemble (WTE) [38, 39] (PTmetaD) was chosen due to its proven ability to fully converge complex conformational free energy surfaces such as those relevant in kinases (including the DFG-flip). Indeed, the PTmetaD approach (both in the standard and WTE variants) has already successfully used to study the conformational dynamics and its associated free energy landscape in many kinases [12, 13, 29, 40, 41] and other flexible proteins [39, 42]. PTmetaD was performed using the software Gromacs 4.5 [43] and the PLUMED plugin [44], using an integration step of 2 fs. The particle mesh Ewald algorithm was used for electrostatic interactions. Temperature coupling was done with the V-rescale algorithm [45]. The WTE allowed the use of a reduced number of replicas compared to standard PTMetaD [12, 39]. An average exchange probability of 24% was obtained using 5 replicas in the temperature range 305–400 K. We used the same four collective variables (CVs) that were used to reconstruct the free energy surface (FES) associated with the DFG flip in Src and Abl [29].

They are shown in S6 Fig and defined in the following (SRC numbering): CV1 is the distance between the centre of mass of Asp_404_ (DFG motif) and Lys_295_. CV2 is the distance between the centre of mass of Phe_405_ and the C_*β*_ of Ile_293_. CV3, is a function of 3 dihedral angles *f* *(* *ϕ*_404_, *ψ*_405_, *ψ*_408_ *)* ranging from 3, when the three dihedral arguments correspond to the DFG-Asp-in position, to 0, when they are in the DFG-Asp-out conformation. CV4 is the distance between the centre of mass of residues Asn_381_, …, His_384_ and residues Ala_408_, …, Ile_411_ of the activation loop, known to form a *β*-sheet in the active conformation. The height of the Gaussians was set at 2.0 kJ/mol with a deposition rate of 1/2000 steps and a bias factor of 5. The Gaussian width used for the CVs was 0.1 for the dihedral similarity (CV3) and 0.3 Å for all the others.

A minimum of 400 ns of sampling per replica in the NVT ensemble were needed to reach full convergence of the free energy. In the case of G250E and T315I, as the convergence was slower than in the other cases, we performed more than 600 ns and 1200 ns of sampling per replica, respectively. The total sampling time amounted to more than 14 *μ*s across all mutants. The free energy surface reconstruction was obtained from the PT-metaD-WTE by reweighting the fixed potential energy bias and using two independent approaches: integrating the bias or using the time independent estimator of Ref. [46]. The convergence of the free energy reconstruction was monitored by integrating the cumulative added bias as a function of time (50 ns intervals) and comparing the reconstruction to that obtained by the time-independent estimator (as shown in S11, S14–S18 Figs). Changes of all the CVs used were also monitored to guarantee that the system diffuses freely in the CV space and is able to visit all the basins several times (see S12 Fig).

The FES were also reprojected as a function of two other CVs describing the conformational change of the A-loop from open to closed. Two path collective variables [47] were built by using the open and closed crystallographic structures of Src (CV1) and Abl (CV2). The reweight was performed by using our python implementation of the approach of Ref. [46] (available on our homepage: https://www.ucl.ac.uk/chemistry/research/group_pages/prot_dynamics/. The entropic contribution to the DFG-in DFG-out free energy difference was computed by linear fitting of the free energy differences as a function of temperature. It has been shown that this approach is more accurate than competing ones. [48]. We also tested the robustness of the estimate with respect to the range of temperatures on both standard PT and WTE-PT (see S8 Fig).

The drug binding free energy surface was calculated using metadynamics with the same software described above. The General Amber Force Field (GAFF) was used for the ligand. The ligand charges were calculated at the HF level using a 631-G* basis set with the Gaussian03 [49] package. QM-level torsional scans with a step of 10 degrees were carried out for the *ca-ca-n-c* and *ca-ca-c3-n3* dihedrals which appeared to have wrong torsional profiles. The profiles with the GAFF force field in vacuum were then fitted to the QM ones to refine the dihedral parameters. As customary in the case of ligand binding [50, 51], we performed a preliminary metadynamics run using sub-optimal geometrical CV to obtain an initial pathway for the setup of the path collective variables (PCV) [47]. We selected 23 frames from the lowest free energy path obtained in the preliminary run and optimized this initial guess using the methodology described by Branduardi et al. [47]. To take into account possible rearrangements, we included the C*α* atoms of the A-loop and of the *α*C-helix in the definition of the PCVs. The 2 PCVs *s* and *z* were used to run a 300 ns metadynamics. The height of the Gaussians was set at 2.0 kJ/mol with a deposition rate of 1/2000 steps and a bias factor of 10. The Gaussian width used for the CVs was 0.1 for *s* and 0.003 for *z*. The free energy corresponding to the last leg of the unbinding mechanism (from the external binding pose to a fully solvated state) was computed again by using Well-tempered metadynamics following the approach of Ref. [52].

### Mutagenesis and ITC Measurements

Src was expressed and purified following the procedure detailed in Ref. [53]. For the first two mutants, an auto-induction protocol was used to reach a good bacterial expression. Thermodynamic binding parameters for the association of imatinib to Src wild type and to the designed mutants were acquired using a VP-ITC microcalorimeter (MicroCal Inc.). The sample solution consisted of 2 ml 0.1 mM protein in MES buffer (pH 6.5) supplemented with 2% DMSO. The ligand solution consisted of 2 ml 1.6 mM imatinib in MES buffer (pH 6.5) with 2% DMSO. Both solutions were degassed for 5 minutes under vacuum. Titrations were conducted at 30°C, consisted of a first control injection of 1 *μ*l followed by 37 injections of 8 *μ*l over 16s, each spaced by an equilibration period of 480s. The samples were stirred at 266 rpm. Raw data were collected and corrected for ligand heats of dilution. A one site binding model was assumed and data were fit using MicroCal Origin software (version 7.0). All experiments were repeated in triplicate.

## Results and Discussion

We ran MD simulations on wild-type (WT) Abl, five clinically-reported drug-resistant mutants of Abl and five WT tyrosine kinases (see Table 1) [29].

**Table 1:** RMSF of the A-loop region and IC_50_ values of imatinib for the studied tyrosine kinases and the designed triple mutants of Src.

TK	A-loop RMSF (Å)	IC_50_ (*μ*M)
Abl	4.2	0.037 [58]
E450K	4.85	2.3 [16]
T315I	2.5	6.4 [59]
H396P	2.15	9 [17]
E279K	2.6	9.9 [16]
G250E	1.8	>20 [16, 54]
Kit	5.5	0.3 [55]
Lck	3.2	9 [56]
Met	2.6	>100 [5]
EGFR	2.5	>100 [57]
Src	2	>100 [5, 57]

The aim is to assess if there is a correlation between the changes in sub-*μ*s dynamics of the TK structure (both in WT proteins and drug-resistant mutants) and the binding affinity of imatinib. We probed the changes in sub-*μ*s dynamics by comparing the root mean square fluctuations (RMSF) of the C_*α*_ averaged over 30ns-long time windows. Among the most common drug-resistant Abl mutants found in relapsing leukemia patients, we selected the gatekeeper mutant (T315I) and four others far from the binding site (allosteric) having a strong impact on imatinib binding (higher IC_50_ values, see Table 1) [16, 17, 54]. Apart from the widely discussed gatekeeper mutant (T315I), the mechanism of resistance of the others is completely unknown.

In addition to Abl and its mutants, we selected a pool of homologous tyrosine kinases that, while sharing the same imatinib binding mode, are inhibited by it with varying strength (see Table 1) [5, 55–59].

**Fig 2:**
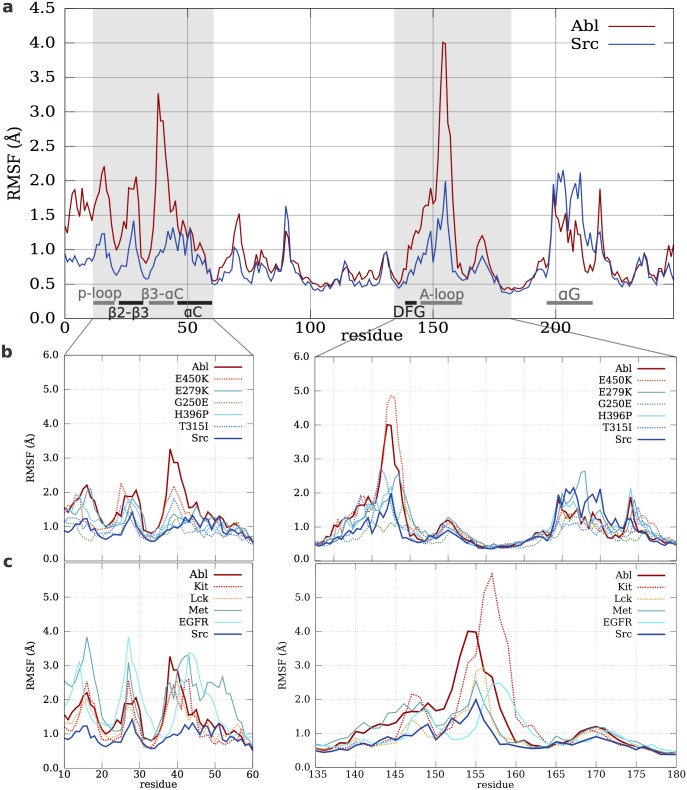
Root mean square fluctuation analysis of TKs and Abl resistant mutants. (a) RMSF of Abl and Src. Fluctuations of the N-lobe (left) and of the A-loop (right) for the Abl mutants (b). and the TKs (c). Shades of red and blue identify strong and weak binders, respectively. Dotted lines are used for clarity.

The RMSFs (Fig 2) show that all the Abl mutants and the imatinib weak-binders TKs (Src, Met and EGFR) are more rigid than WT Abl [29]. The regions most affected are the functionally-relevant regions of the N-lobe (p-loop, *α*C-helix and the *β*3-*α*C loop), the A-loop and, to a lesser extent, the *α*G-helix, whose flexibility profiles are more “Src-like” [29]. An analysis of the diffusion of the dynamics in the space defined by the first two principal component vectors of Abl is also consistent with the rigidification of the mutants (see S9 Fig).

The DFG-out states, with the notable exception of G250E and T315I, are even more rigid, indicating a probable entropic penalty with respect to the DFG-in states (see S7 Fig). The *β*3-*α*C loop, which is known to play a role in the allosteric activation of Abl by the SH2 domain [40, 60] is the most affected element.

The A-loop is more rigid in most mutants and in all the weak-binders group (Src, Met and EGFR), while it is comparatively more flexible in the strong imatinib binders (Abl, Kit and Lck). A-loop flexibility and IC_50_ are anti-correlated (Table 1). Finally, the *α*G-helix, an hotspot for allosteric regulation in TKs [20, 61], is very flexible in the weak-binding group and in E279K resistant mutant (see S2 Fig) and can experience unique conformational changes (see S2 Fig). The clinical drug-resistant mutants of Abl [16] are prevalently located in regions of the structure whose flexibility varies across weak and strong-binding TKs (see S1 Fig). This should not be surprising, as the observed flexibility changes affect regions that are either directly involved in the binding of type II inhibitors (the DFG-motif, A-loop [25] and p-loop. [62], see S1 Fig)) or allosterically connected to the former (*α*C-helix, *α*G-helix, *β*3-*α*C loop) [20, 40]. Thus, the change in the sub-*μ*s dynamics of these regions could affect the entropic contribution to the relative stability of the inactive and active states, in turn decreasing the binding affinity of imatinib and other type II inhibitors that bind to an inactive state.

### Site-Directed Mutagenesis

To understand whether inducing a change in the flexibility of relevant structural motifs is sufficient to increase the drug sensitivity, we designed a number of Src KD mutants and measured the changes in K_d_.

We engineered mutations in four different locations, the P-loop (Q275), the *β*3-*α*C loop (P299) the A-loop (Q420) and the *α*G-helix (V461), to the equivalent residues in Abl and Lck. The residues to be substituted were chosen in the functionally-relevant regions showing a major variation in the sub-*μ*s dynamics, between the weak and strong imatinib binders. We first performed MD simulations and compared the flexibility of various mutants to that of Src WT. Based on the RMSF we choose three candidates for the experiments. The first bears Q275A, P299Q and Q420E substitutions (Src to Lck), the second Q275G, P299E and Q420A (Src to Abl) and a third one with Q275A, P299Q and V461S (Src to Abl).

The RMSF shows an increased flexibility in the N-lobe (p-loop, *β*3-*α*C loop and *α*C helix) compared to Src WT (see S3 Fig). For the mutant Q275G/P299E/Q420A, also an increase in A-loop flexibility was identified. In the Q275G/P299E/V461S mutant was introduced a mutation on the *α*G-helix, that has provoked a suppression of the fluctuations in this area, resembling the *α*G-helix of Abl. The K_d_ appear to be anti-correlated with the RMSF of the A-loop (Table 2). As a cross-validation, when a chimeric protein with the entire *α*C-helix of Abl was produced, we observed a general suppression of the dynamics and an increased K_d_ of 14 *μ*M. At difference with previous studies reporting similar K_d_ [26], the mutated residues are far from the binding site and do not interact with the drug.

**Table 2:** K_d_ for the Src mutants, Abl and Src WT. The RMSF values have been averaged from MD simulations.

Protein	A-loop RMSF (Å)	K_d_ (*μ* **M**)
Abl WT	4.2	0.08 [26]
Q275G/P299E/V461S	1.4	6.2
Q275G/P299E/Q420A	2.5	7.3
Q275A/P299Q/Q420E	1.8	8.5
Src WT	2	12
*α*C chimera	0.3	14

### Conformational Dynamics

If the correlation between changes in the sub-*μ*s dynamics and imatinib affinity observed in the MD simulations and validated by experiments is due to entropic effects it must be reflected in the conformational free energy landscape (and in the entropic contribution to the relative stabilities of the DFG-in and -out states). As discussed above, Imatinib binds to an inactive state in which both the conformation of the DFG motif and that of the A-loop are important. In non-phosphorylated TKs the active-like “open” state in which the A-loop is extended, is marginally populated [12, 63, 64]. The DFG-out inactive state has a significant thermodynamic penalty and (according to the observed changes in the sub-*μ*s dynamics) is in most cases entropically disfavored. In both inactive states the delicate balance between entropic and enthalpic contributions are crucial in defining their stability with respect to the open, DFG-in active state. This balance can be easily upset by drug-resistant mutations either directly or through the allosteric network. Thus a mutation affecting the entropy / enthalpy balance of the active and inactive states could easily shift the kinase towards active state, decreasing the binding affinity of type II inhibitors. Indeed, the stabilization of the active (extended A-loop) conformation has been experimentally observed for many TK gatekeeper mutants (including Abl T315I) [10, 11, 13, 65].

**Fig 3:**
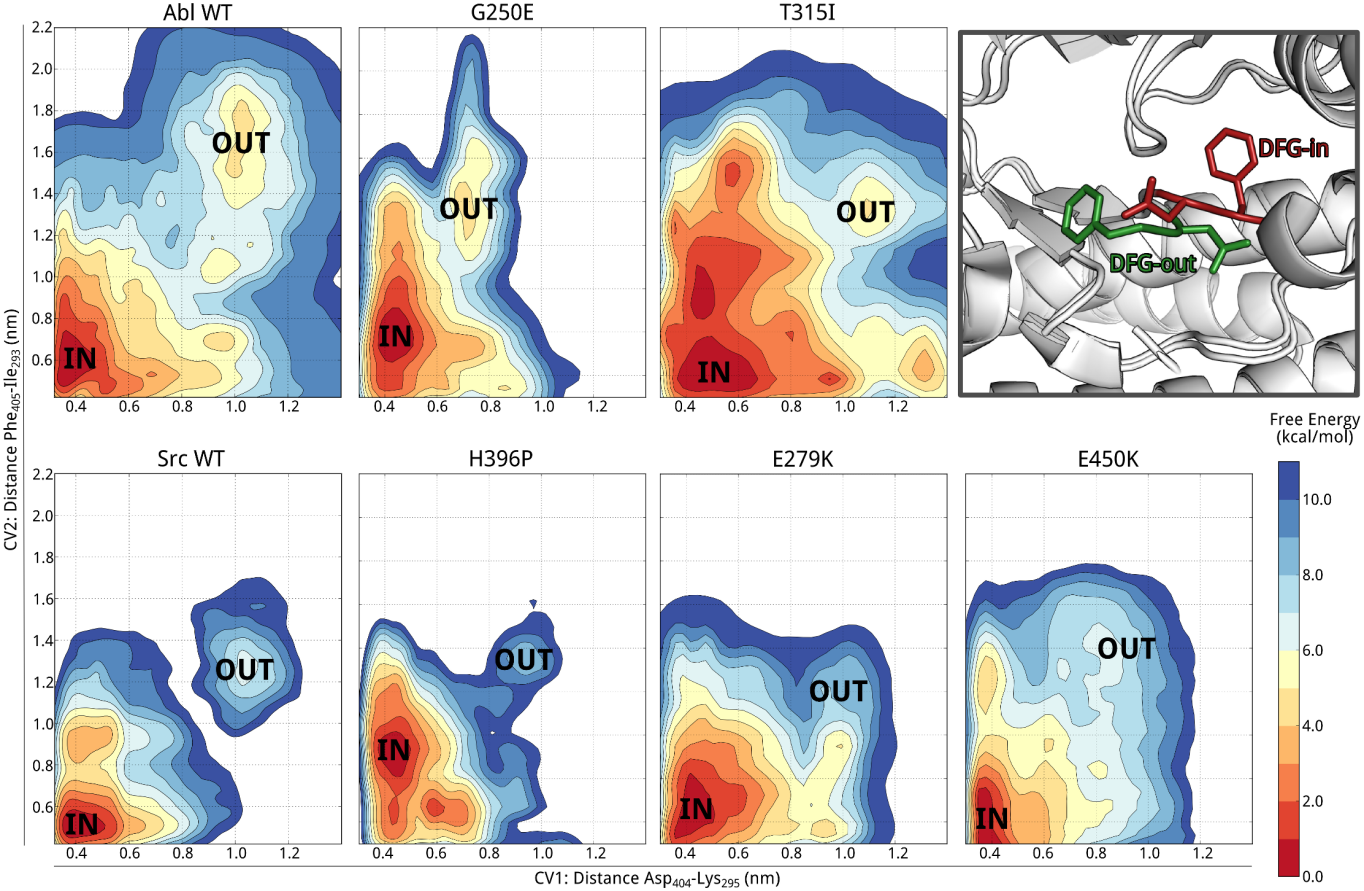
Free energy of the DFG flip transition. Free energy surfaces of Abl, Src (adapted from Ref. [29]), and Abl drug-resistant mutants projected on the distances between DFG Asp_404_ and Lys_295_ (CV1) and DFG Phe_405_ and Ile_293_ (Leu_137_ in Src) (CV2). The free energy minima corresponding to DFG-in conformations are labeled “IN”, while “OUT” correspond to DFG-out conformations. The contour lines are drawn every 1 kcal/mol.

To further investigate this issue, we performed large-scale multiple-replica PT-MetaD simulations and computed the fully converged conformational free energy landscape associated with the DFG-flip of the five drug-resistant mutants. In Fig 3 we report the FES projected as a function of two collective variables (CVs) that distinguish the DFG-in from the DFG-out state [29], namely the distance between the DFG Asp_404_ and Lys_295_ and between the DFG Phe_405_ and Ile_293_ (Src numbering). As expected, for all mutants the global minimum of the FES corresponds to the DFG-in state (basin “IN” in Fig 3). The mutants also explore the DFG-out state (basin “OUT”), which in most cases corresponds to a well-defined metastable minimum. All mutations alter significantly the conformational free energy landscape. The free energy penalty of the DFG-out state increases going from Abl WT and G250E (ΔG of 4 ± 0.5 kcal/mol) to E450K (6 kcal/mol), H396P and E279K (7 ± 0.5 and 8 ± 0.5 kcal/mol). The gatekeeper T315I and (to a lesser extent) G250E are significant exceptions as the ΔG associated to their DFG-flip is very close to that of the WT. The increased thermodynamic penalty is due to a net entropic loss of the DFG-out state, as shown in Table 3 (the DFG-in/-out free energy differences as a function of the temperature are reported in S8 Fig). Again T315I and G250E are special cases as there is an entropic gain for the DFG-out state. The entropic contributions to the DFG flip are in excellent agreement with the observed changes in the sub-*μ*s dynamics.

**Table 3:** Entropy and Enthalpy contributions to the DFG-in DFG-out flip as obtained from the linear regression of the Free Energy as a function of temperature.

TK	Δ*H* (kcal/mol)	*T*Δ*S* (kcal/mol)
Abl	-3.56	-8.01
E450K	-2.66	-8.96
T315I	17.93	13.06
H396P	-7.61	-14.51
E279K	1.02	6.9
G250E	43.69	39.79
Src	-9.73	-15.28

Indeed in H396P and E279K, where the A-loop is significantly more rigid, the flip has a larger free energy penalty. In the cases of G250E and the T315I gatekeeper mutant the penalty for the flip is comparable to that of the WT, and in agreement with the observed increased flexibility of the N-lobe and A-loop in the DFG-out state, there is an entropic gain associated with it. In both cases the DFG-out has a peculiar geometry where both Asp_404_ and Phe_405_ point outwards (S4 Fig) and an open active-like extended A-loop conformation is observed.

**Fig 4:**
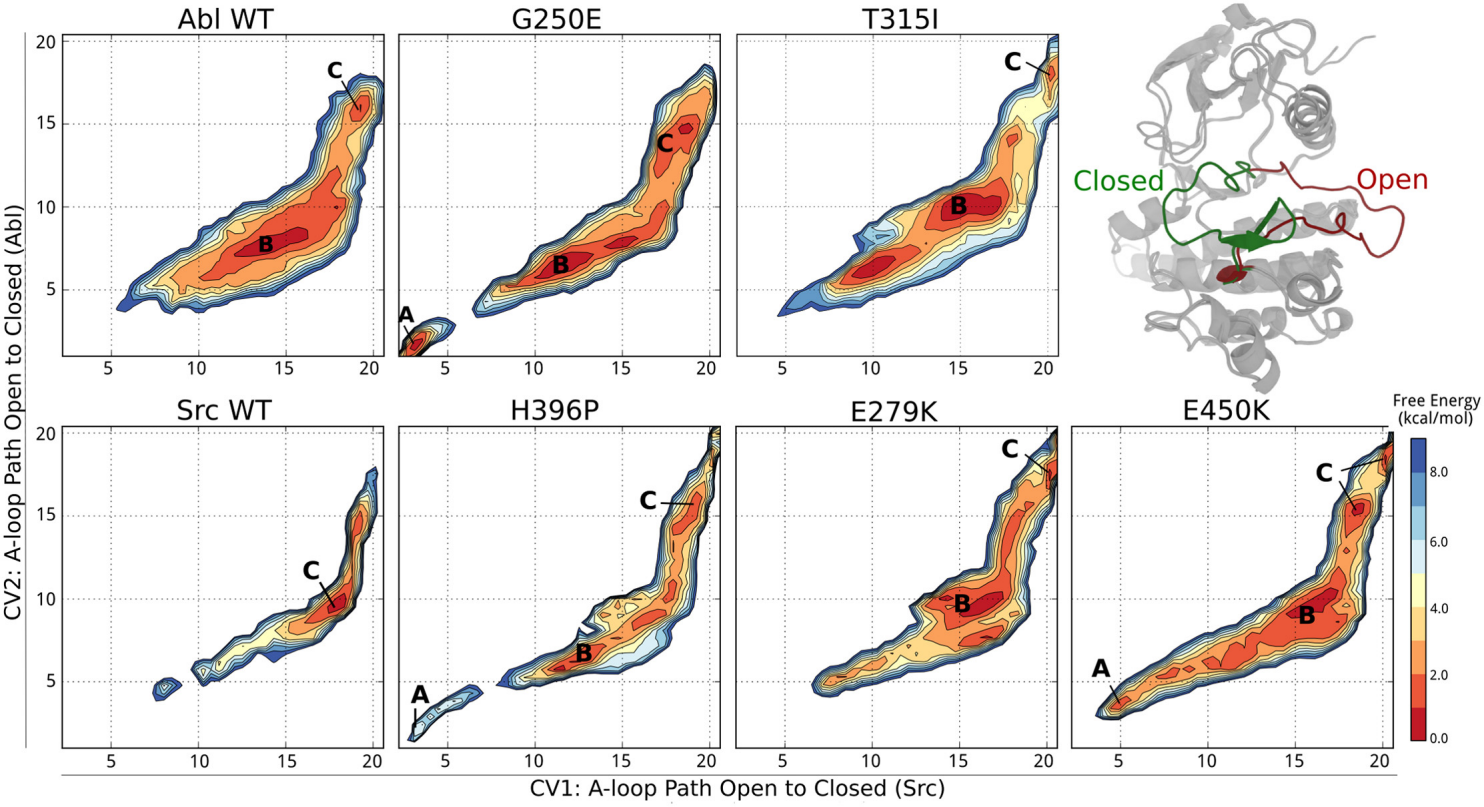
Free energy of the A-loop opening. Free energy surfaces of Abl, Src, and drug-resistant mutants projected on the optimal path describing the conformational change of the A-loop from open to closed in Src (CV1) and Abl (CV2). The free energy minima corresponding to an extended A-loop active-like conformation are labeled “A”, “B” is used for A-loop semi-closed (inactive) conformations and “C” for fully closed A-loop conformations. The contour lines are drawn every 1 kcal/mol.

To quantify this aspect, we analyzed how the population of the “open” state is altered by the mutations. We re-projected the free energy surfaces (FES) as a function of path variables [47] describing the opening of the A-loop (Fig 4) with respect to Src (CV1) and Abl (CV2).

Abl, Src and E279K mainly populate semi-closed and closed conformations (basin B and C), while G250E, T315I and to a lesser extent E450K, H396P show a minimum in correspondence of the open A-loop (basin A, CV1 < 6). The A-loop in these and other mutants tends to form a second helix turn as in Src (S5 Fig). The residues involved are Asp_391_ to Ala_395_, which in the case of H396P are right before the mutation. The formation of this helix has been shown to stabilize the extended A-loop active state in TKs [10] and is also involved in the mechanism of oncogenic mutations [7, 12]. The stabilization of the active state is known to weaken the binding of ATP-competitive inhibitors to oncogenic mutants of EGFR [10, 12] and in the phosphorylated form of Abl [25, 26]. In the case of T315I the observed stabilization of the active state is in agreement with multiple experimental observations [10, 11, 13, 65].

Finally, we also observe a loss of structural integrity of the *α*C and *α*G helices in the DFG-out state of most mutants (S4 Fig) in agreement with previous proposals stating that conformational transitions in kinases are accompanied by local unfolding of secondary structural elements [66, 67].

### Binding Mechanism

**Fig 5:**
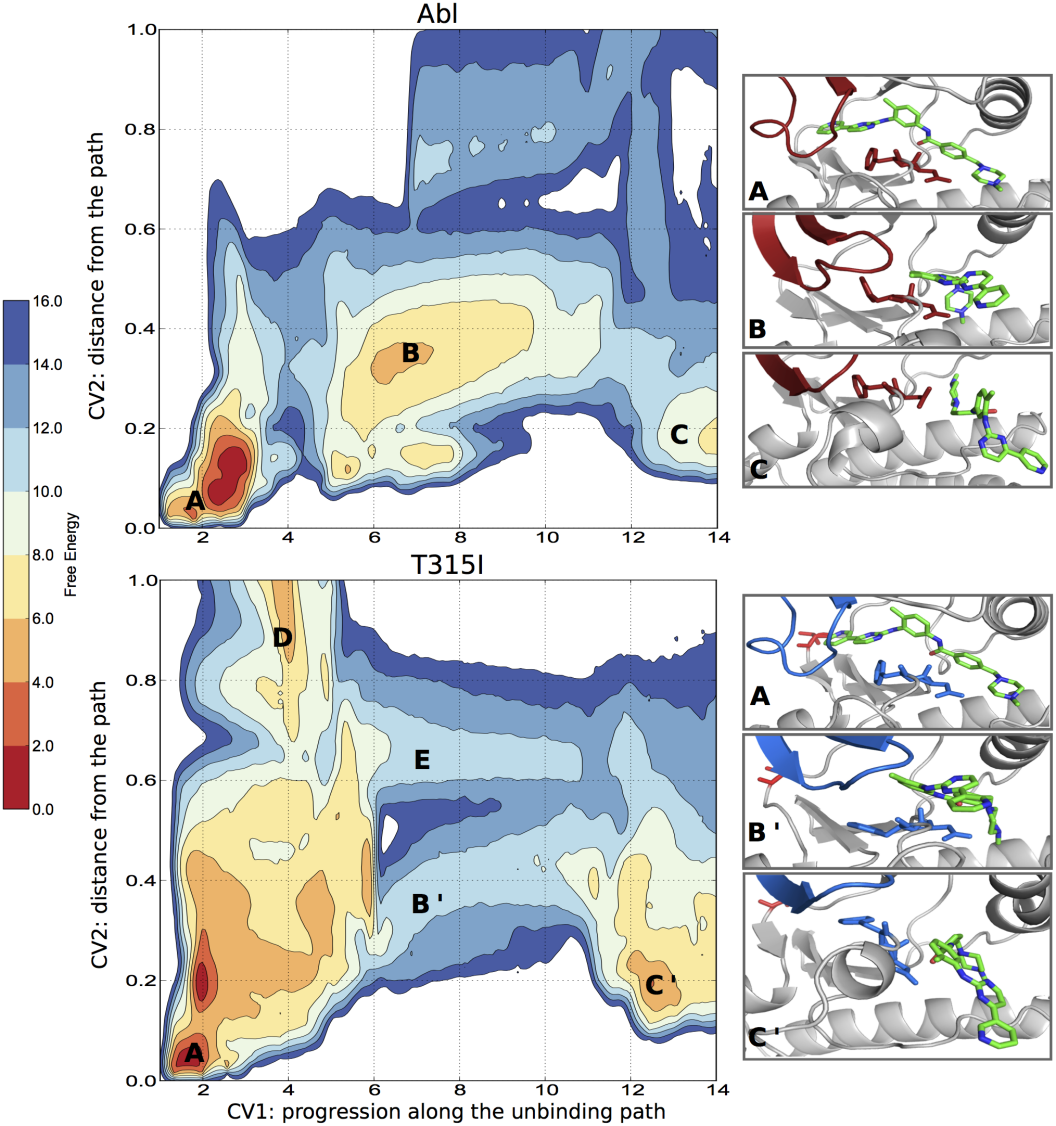
Free energy of imatinib (un-)binding to Abl and to the T315I ‘gatekeeper’ mutant. Free energy surfaces associated to the binding of imatinib to WT Abl (top panel) and the T315I Abl “gatekeeper” mutant (bottom panel). The deepest energy minima correspond to the crystallographic binding pose and are labeled A. On the way out, B and B’ correspond to an intermediate state (metastable in WT Abl) where imatinib is in between the DFG and the *α*C helix. States C and C’ correspond to the “external binding pose”. Interestingly in Abl T315I there are two exit channels and both have an higher barrier than in the WT. The contour lines are drawn every 2 kcal/mol.

Our results appear to be consistent with a significant impact of the resistant mutations on two different conformational changes: the DFG flip, disfavoring the drug-binding conformation (mainly mutants with rigidified A-loop and N-lobe), and the opening of the A-loop, possibly favoring the locking of the kinase in an active form. However, from the simulations on the unbound kinases, we cannot rule out an effect of the gatekeeper mutation on the proposed “induced-fit” mechanism [14]. Indeed, the predicted ΔG associated to the DFG-flip of T315I, which is very close to that of the WT, leaves two hypotheses open. Either the weaker binding of imatinib is due to the observed stabilization of the extended A-loop active state, or to the suppression of an induced fit effect, possibly acting on the p-loop conformation. To clarify this point and shed more light on the binding mechanism itself, we have computed the binding free energy of imatinib to Abl WT and Abl T315I along a physical association pathway. The use of such an approach, albeit it is significantly more expensive than an “end-point” free energy calculation (e.g. thermodynamic integration) has the advantage of reporting on free energy barriers and thus on the binding and unbinding kinetics. As expected, the crystallographic binding pose corresponds in both cases to the deepest minimum. Starting from that pose, the bidimensional (un-)binding free energy profiles show a substantial difference in imatinib’s mechanisms of binding to Abl WT and T315I (Fig 5). In the WT kinase the barrier to unbinding is lower (in agreement with the observed differences in unbinding kinetics) and there is one main exit path. It is also interesting to note that we find an “external binding pose” from which the inhibitor slides to its final crystallographic binding pose. In the external binding pose the DFG motif is already in the “out” position. In T315I we observe two different unbinding / binding pathways (B’ and E, Fig 5 and S10 Fig) somewhat similar to previous proposals. [68] In both of them the p-loop has a significant role. A pose similar to the external binding pose observed in the WT kinase is present (C’ in Fig 5) but it is slightly shifted towards the *α*C helix and more stable than in the WT. Thus the gain in free energy from the external binding pose to the final (crystallographic) pose is lower, in agreement with the observed increase of the IC_50_. When the unbinding free energy from C to a fully solvated state is accounted for (see S13 Fig), the overall free energy difference from the unbound fully solvated state to the crystallographic pose is around 13 kcal ± 2 (the larger convergence error is due to the algorithms used). When the free energy penalty of the DFG flip (≃ 4 kcal) is subtracted, we get 9 kcal ± 2, in agreement with the calorimetric and IC_50_ experimental data. It is thus clear that the effect of the T315I gatekeeper mutation is dual. It both affects the binding mechanism and stabilizes the active, extended A-loop conformation.

### Conclusions

We have studied the interplay between sub-*μ*s dynamics and conformational dynamics in TKs, and how these are influenced by drug-resistant mutations impacting Type-II inhibitors binding. Overall, our simulations show that drug-resistant mutations have a significant effect on both the sub-*μ*s dynamics and the conformational free energy landscape. They affect the energy and the population of the DFG-out inactive state and of the open A-loop active-like state. Since type II inhibitors bind to the inactive kinase, a more accessible DFG-out state leads to a stronger binding of imatinib and other type II inhibitors. On the contrary, a more populated active-like state, in which the A-loop is elongated, increases the affinity towards ATP and disfavors type II inhibitors.

The selected drug-resistant mutants fall in two partially overlapping categories: those that have a significantly higher free energy penalty for the DFG-out state (E279K, H396P) and those (G250E, E450K, T315I) that populate the A-loop open active-like state. What is more the T315I gatekeeper mutant has a significant impact on the binding mechanism itself and on the binding kinetics.

The mutations affect the free energy differences associated to the conformational changes mainly by changing the sub-*μ*s dynamics and consequently the entropy / enthalpy balance of the different states. Thus, relative short MD simulations, by revealing changes in the sub-*μ*s dynamics, might be used to predict the impact of new mutations on Imatinib resistance.

The important role played by the entropic penalty of the DFG-out state must also be kept in mind when comparing low temperature and room temperature experiments. On the whole, we characterized the mechanism of action of several drug-resistant mutants of Abl. We have shown the link between fast and slow dynamics in these complex systems, providing a deeper understanding of the thermodynamics, kinetics and allosteric regulation of type II inhibitor binding TKs. In perspective, our results could help the design of fast and predictive computational approaches to predict the effect of yet unknown mutations of TKs.

## Supporting Information

S1 Fig Correlation between the structural position of resistant mutants of Abl and their impact on imatinib IC_50_. (a) Binding modes of Type I and Type II kinase inhibitors. The ATP binding site is divided into two sub regions, the ATP pocket (in pink) is occupied by both Type I and II binders, while the allosteric pocket (in blue) is occupied just by Type II inhibitors, like imatinib. The A-loop is colored in yellow and the DFG in green. (b) IC_50_ for each mutation of Abl found in the work of Azam et al. and (c) corresponding position in the Abl structure. Mutations localized in flexible regions are colored in orange, the ones lying in rigid regions in green.
(PDF)

S2 Fig *α*G-helix dynamics and typical conformational changes. Fluctuations of the *α*G-helix region for all the Abl drug-resistant mutants (a) and all the tyrosine kinases (b) under study. Shades of red have been used to identify strong imatinib binders, while shades of blue identify weak binders. Solid and dotted lines have been used for clarity. (c,d) Different *α*G-helix conformations peculiar of the Src kinase.
(PDF)

S3 Fig Experimental data retrieved for the engineered mutants of Src. (a) RMSF of the N-lobe (left panel) and the A-loop (right panel) regions for the engineered Src mutants. (b) HisTrap and S75 columns chromatogram; the protein of interest is the more intense UV signal. (c) Gels showing the obtained purified mutants. (d) Titration calorimetry experiments of Src WT and of the designed mutants.
(PDF)

S4 Fig Conformations of resistant mutants of Abl sampled during PTmetaD simulations. DFG-out state of G250E (a) and E450K (b) with a peculiar out-out geometry. Unfolding event of *α*C-helix and *α*G-helix for G250E (c), E279K (d), H396P (e,f) and E450K (g).
(PDF)

S5 Fig Characteristic helix turn formation in the A-loop of the resistant mutants of Abl and theory scheme. In the mutants the A-loop forms a second helix turn, characteristic of the A-loop of Src, shown in (a). The A-loop conformations of the mutants have been shown as follow: G250E (b), E279K (c), H396P (d), E450K (e) and T315I (f). A schematic representation of the proposed resistance mechanism is shown in (g). The destabilization of the DFG-out inactive state plays a prominent role.
(PNG)

S6 Fig Collective variables (CVs) used in the PTmetaD-WTE calculations. Src numbering is used throughout, with the exception of residue L317, that corresponds to residue I293 in Abl (PDB:G1T numbering used). The dihedral angle combination f(*ϕ*_404_, *ψ*_405_, *ψ*_408_) (CV3) is shown on the right.
(PDF)

S7 Fig Root mean square fluctuation analysis of DFG-in and DFG-out conformations. RMSF profiles of DFG-in (in orange) and DFG-out (in green) states for Src, Abl and the resistant mutants of Abl.
(PDF)

S8 Fig Entropic and enthalpic contributions to the DFG flip. Linear regressions of the PTmetaD free energies as a function of temperature. Entropy and Enthalpy contributions have been obtained for Src, Abl and all the resistant mutants.
(PNG)

S9 Fig Principal component analysis. PCA analysis for Abl, Src and the resistant mutants of Abl. The trajectories of Src and of the resistant mutants of Abl have been projected along the first (hinge motion) and second (lobe twist) eigenvector of Abl.
(PDF)

S10 Fig Structures describing the unbinding process of imatinib in T315I mutant. States D and E corresponding to the alternative unbinding path of imatinib in the T315I mutant shown in Fig 5 of the main text.
(PDF)

S11 Fig Monodimensional profile of the free-energy convergence. Typical convergence of the PTmetaD calculations. Projection of the free-energy of H396P along CV1 (distance Asp404-Lys295) and of E450K along CV2 (distance Phe405-Ile293), calculated on the final 200 ns of run, at intervals of 40 ns. The dotted lines correspond to the reweight performed on both simulations.
(PNG)

S12 Fig Diffusion of the collective variables in the PTmetaD simulations. Values of the CVs used for the PTMetaD versus simulation time. In accordance with the behaviour expected, the system diffuses freely in the CV space and visits all the basins within the threshold energy multiple times.
(PDF)

S13 Fig Free-energy profile of (un-)binding of imatinib in Abl WT. Monodimensional profile of unbinding for Abl WT along the distance between the center of mass of residues Asp404-Leu322-Val323 and Imatinib. The state C corresponds to the external binding pose of Imatinib, while in state D imatinib is unbound and fully solvated.
(PDF)

S14 Fig Free-energy surface convergence of G250E. Bidimensional profiles of G250E along CV1 and CV2 calculated at intervals of 20 ns in the last 160 ns of simulation time. The very small changes in the FES and the consistency of the relative ΔGs gives a good indication of the sampling convergence.
(PDF)

S15 Fig Free-energy surface convergence of T315I. Bidimensional profiles of T315I along CV1 and CV2 calculated at intervals of 20 ns in the last 160 ns of simulation time.
(PDF)

S16 Fig Free-energy surface convergence of E450K. Bidimensional profiles of E450K along CV1 and CV2 calculated at intervals of 20 ns in the last 160 ns of simulation time.
(PDF)

S17 Fig Free-energy surface convergence of H396P. Bidimensional profiles of H396P along CV1 and CV2 calculated at intervals of 20 ns in the last 160 ns of simulation time.
(PDF)

S18 Fig Free-energy surface convergence of E279K. Bidimensional profiles of E279K along CV1 and CV2 calculated at intervals of 20 ns in the last 160 ns of simulation time.
(PDF)
