# Supplementary figures and images for: Towards a Molecular Understanding of the Link between Imatinib Resistance and Kinase Conformational Dynamics
Source: PLoS Comput Biol. 2015 Nov 25;11(11):e1004578. doi: 10.1371/journal.pcbi.1004578 (PMC4659586; doi:10.1371/journal.pcbi.1004578)

a

Type I

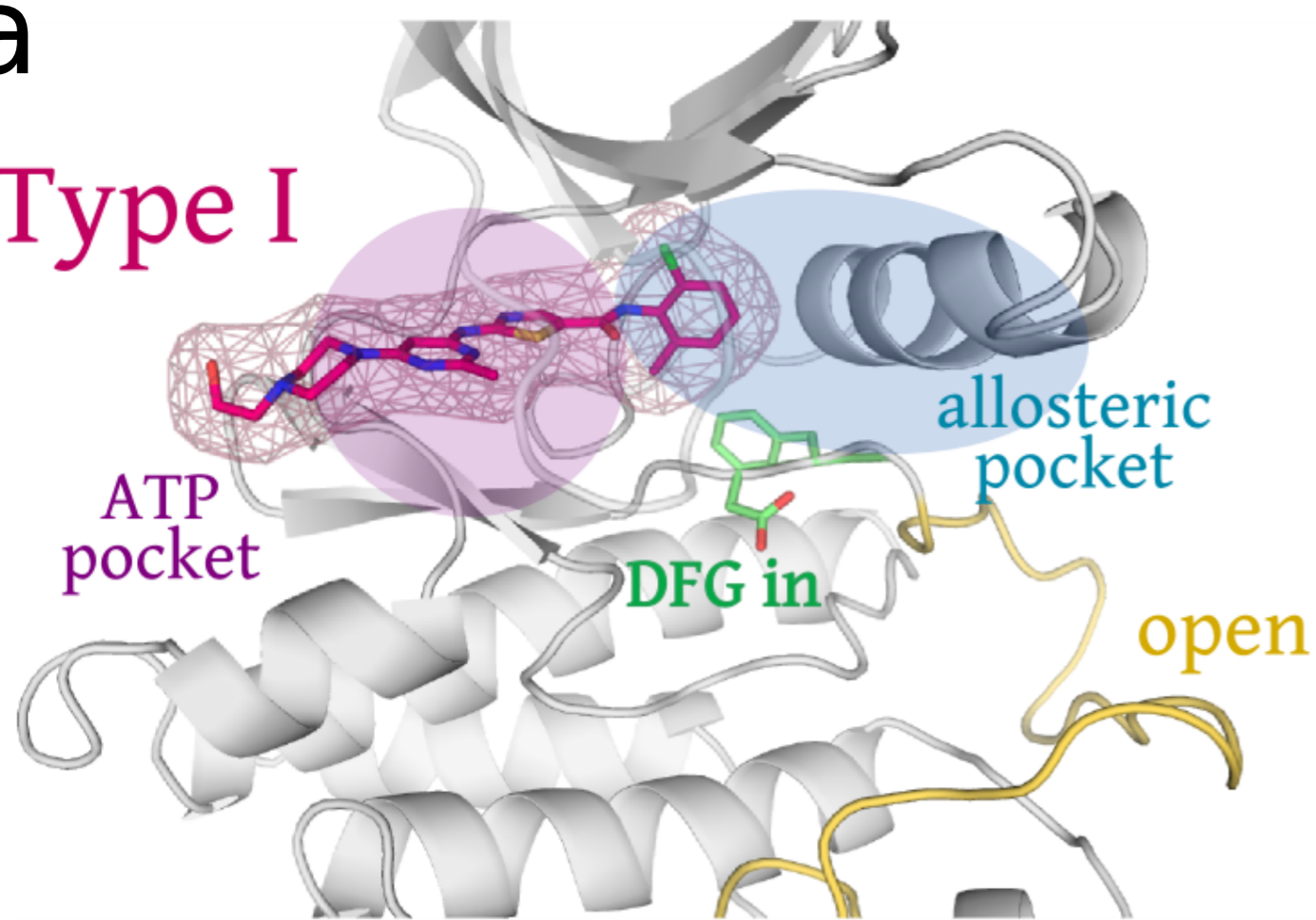

Type II

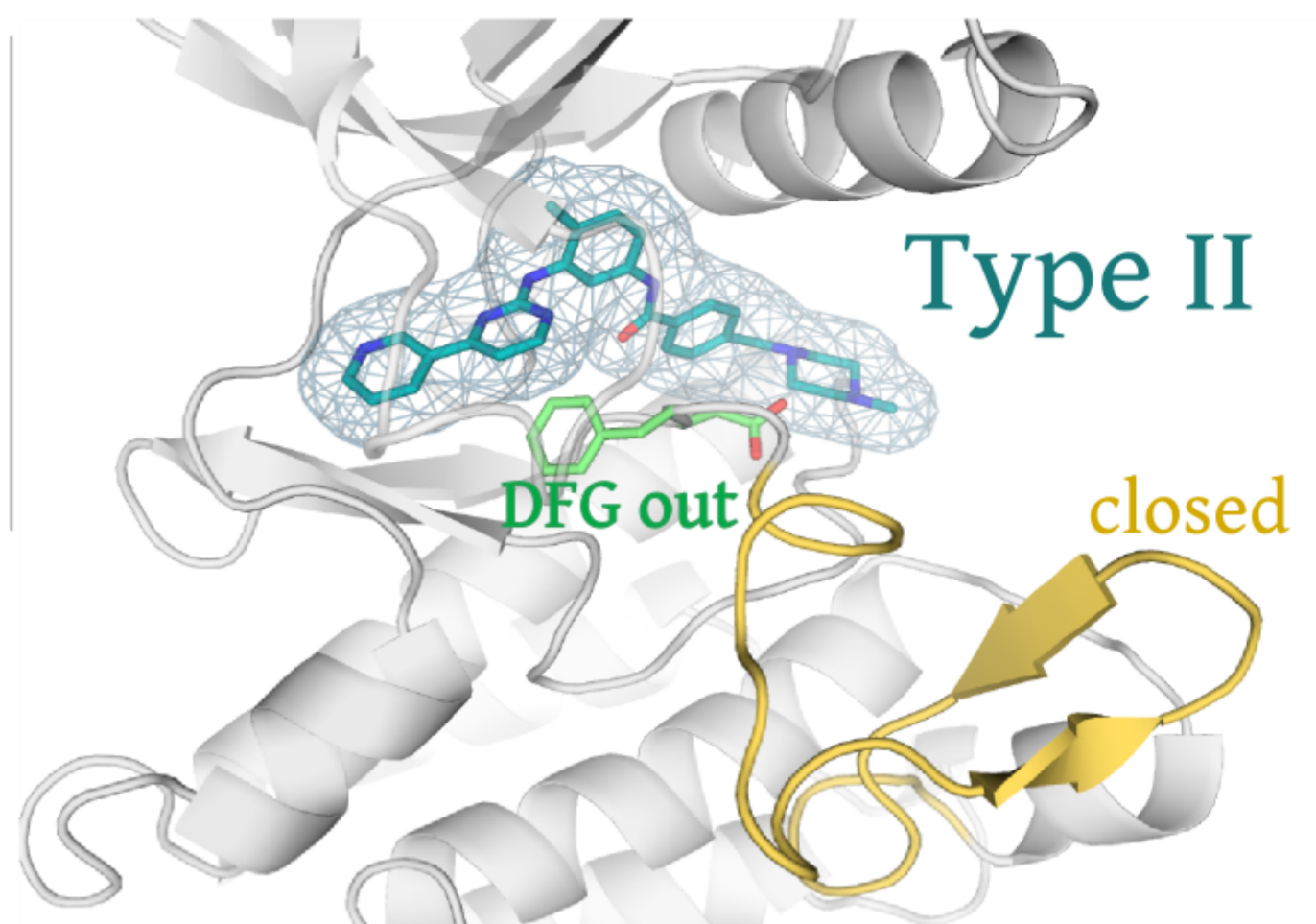

b

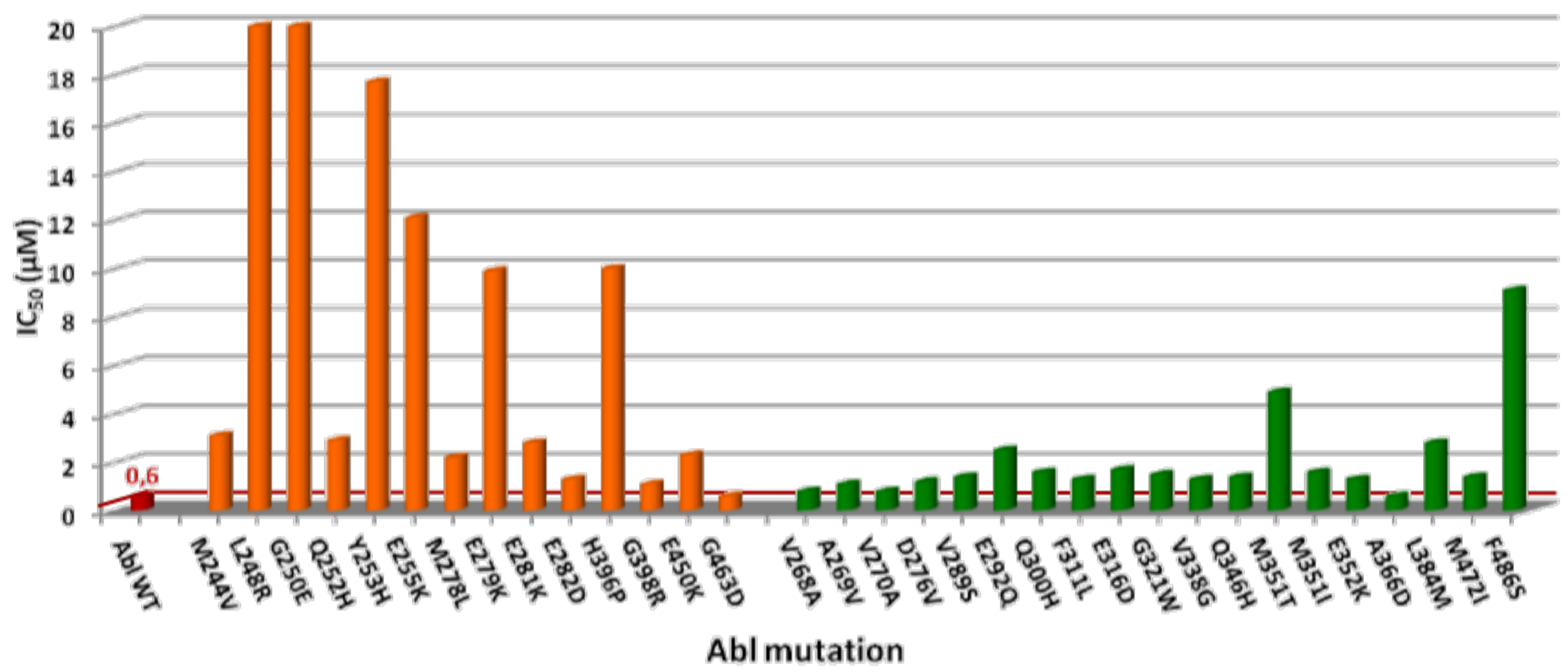

c

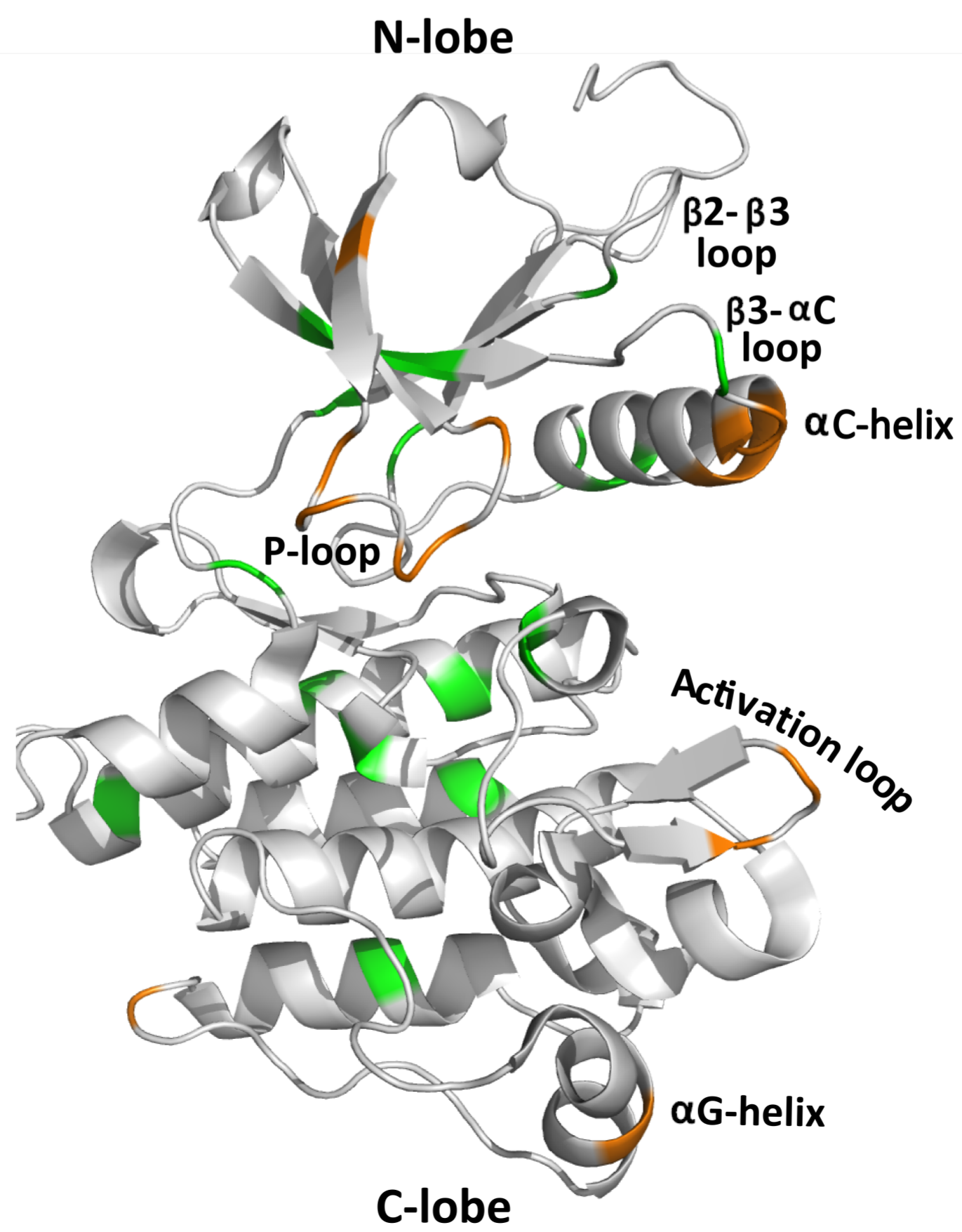

Supplement: S1 Fig — (a) Binding modes of Type I and Type II kinase inhibitors. The ATP binding site is divided into two sub regions, the ATP pocket (in pink) is occupied by both Type I and II binders, while the allosteric pocket (in blue) is occupied just by Type II inhibitors, like imatinib. The A-loop is colored in yellow and the DFG in green. (b) IC50 for each mutation of Abl found in the work of Azam et al. and (c) corresponding position in the Abl structure. Mutations localized in flexible regions are colored in orange, the ones lying in rigid regions in green. (PDF) [file pcbi.1004578.s001.pdf]

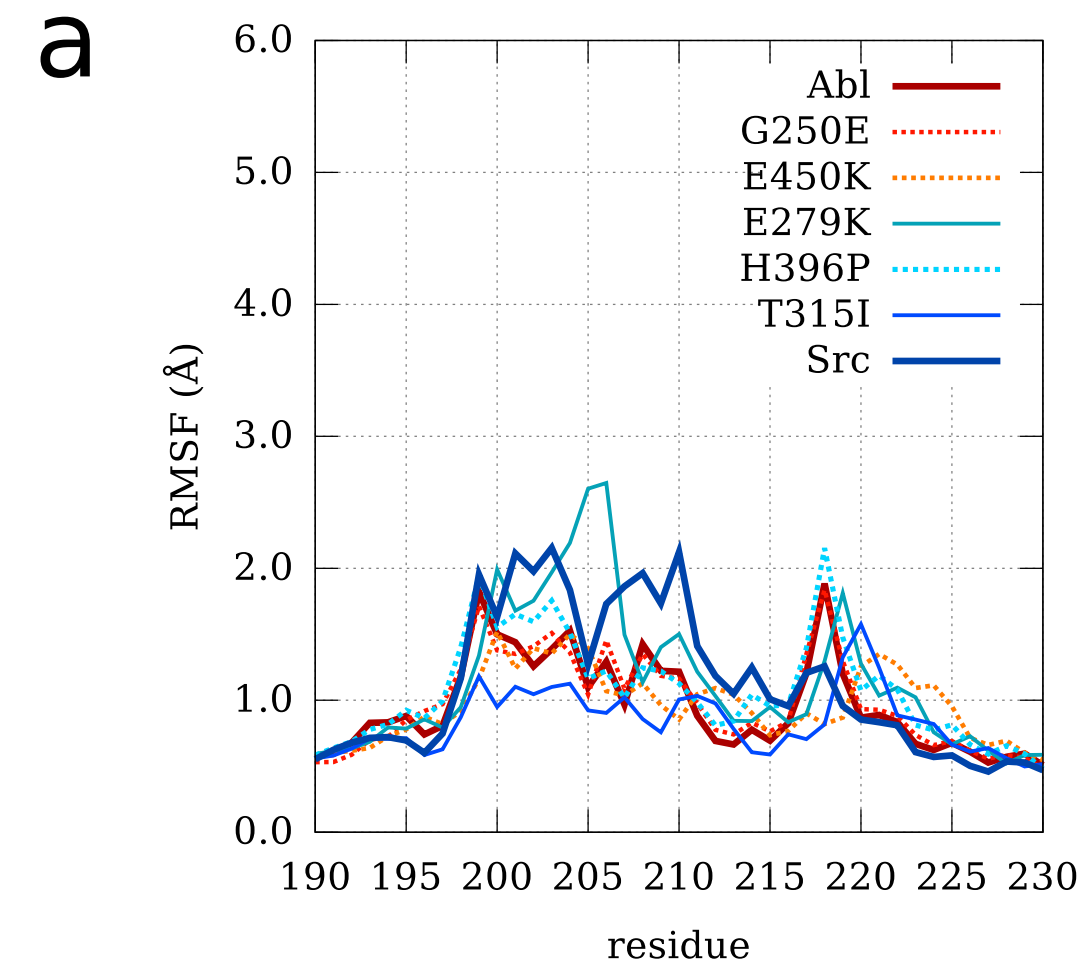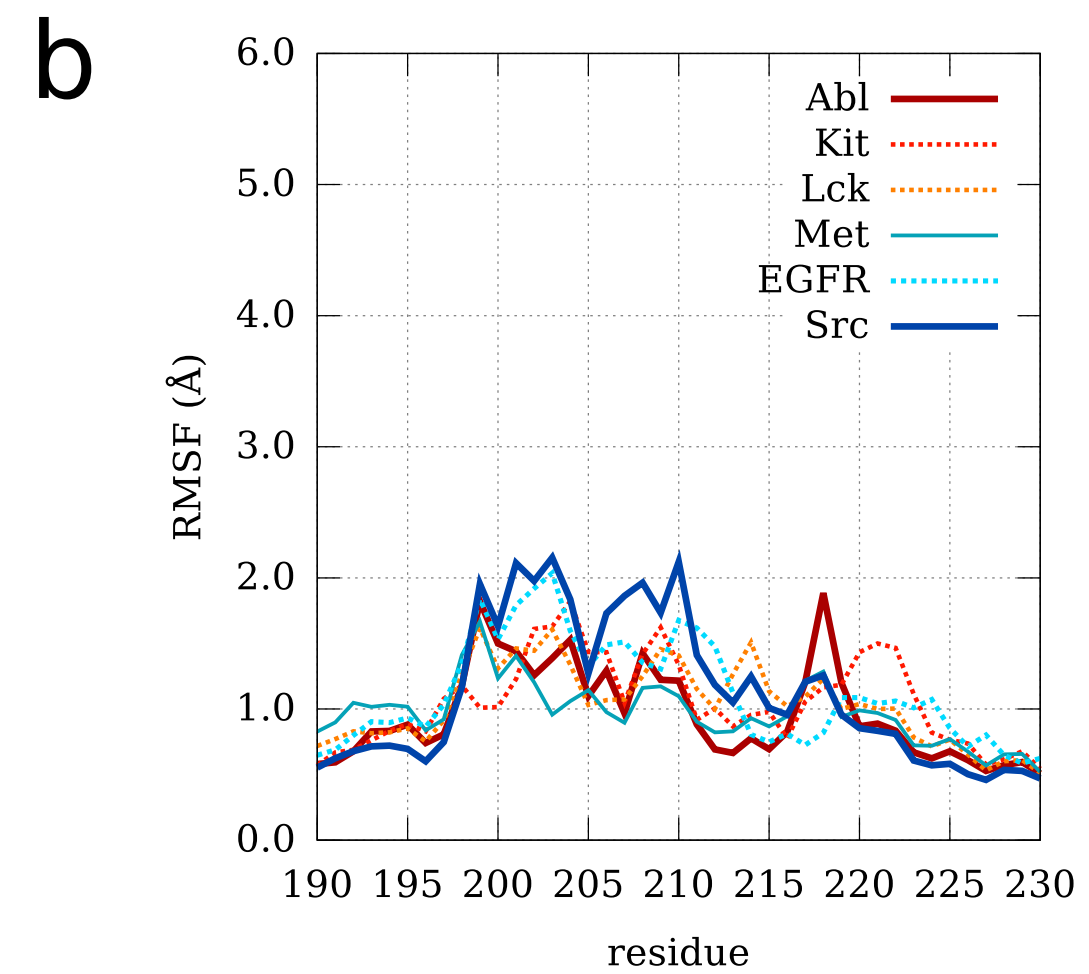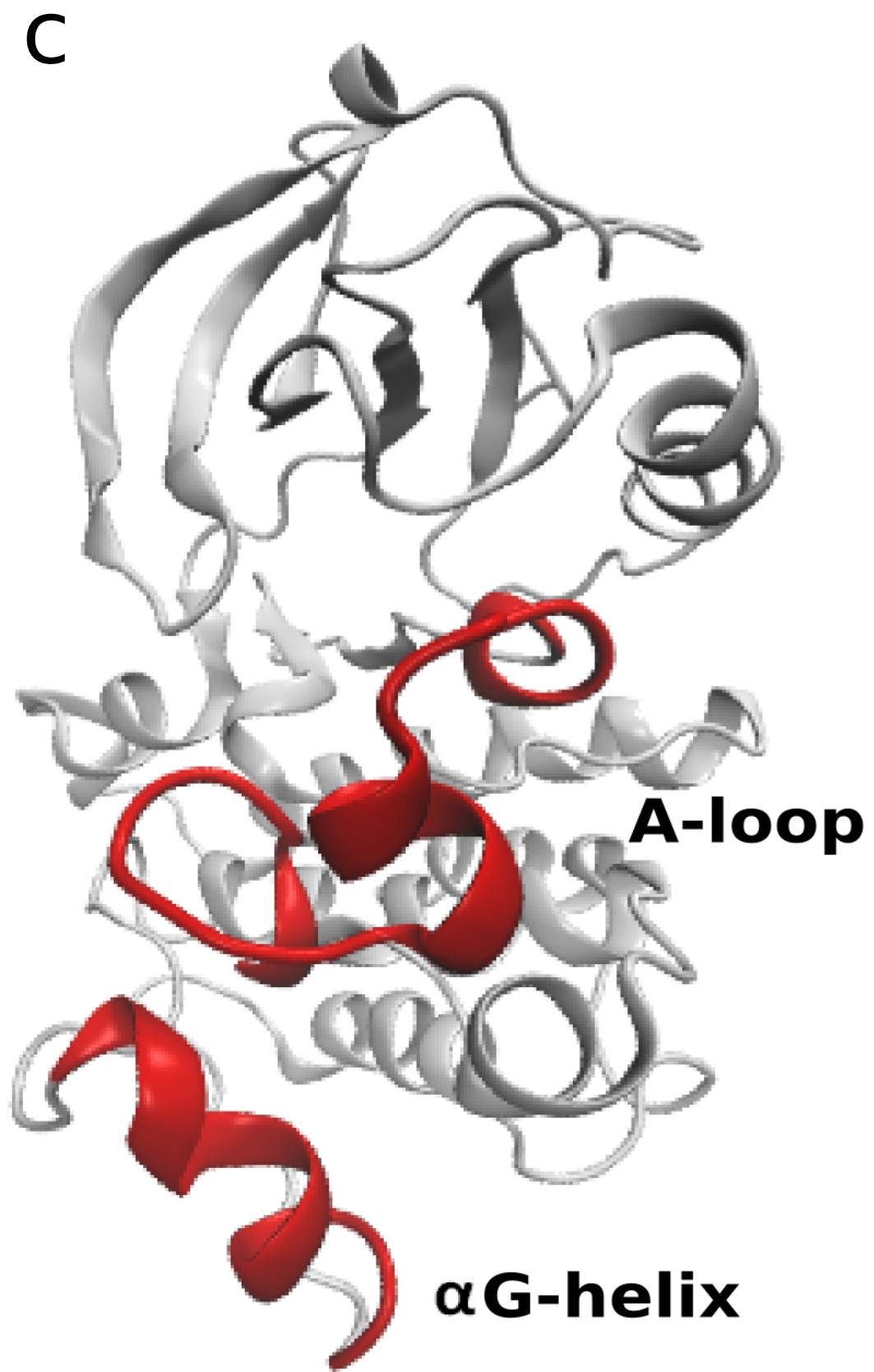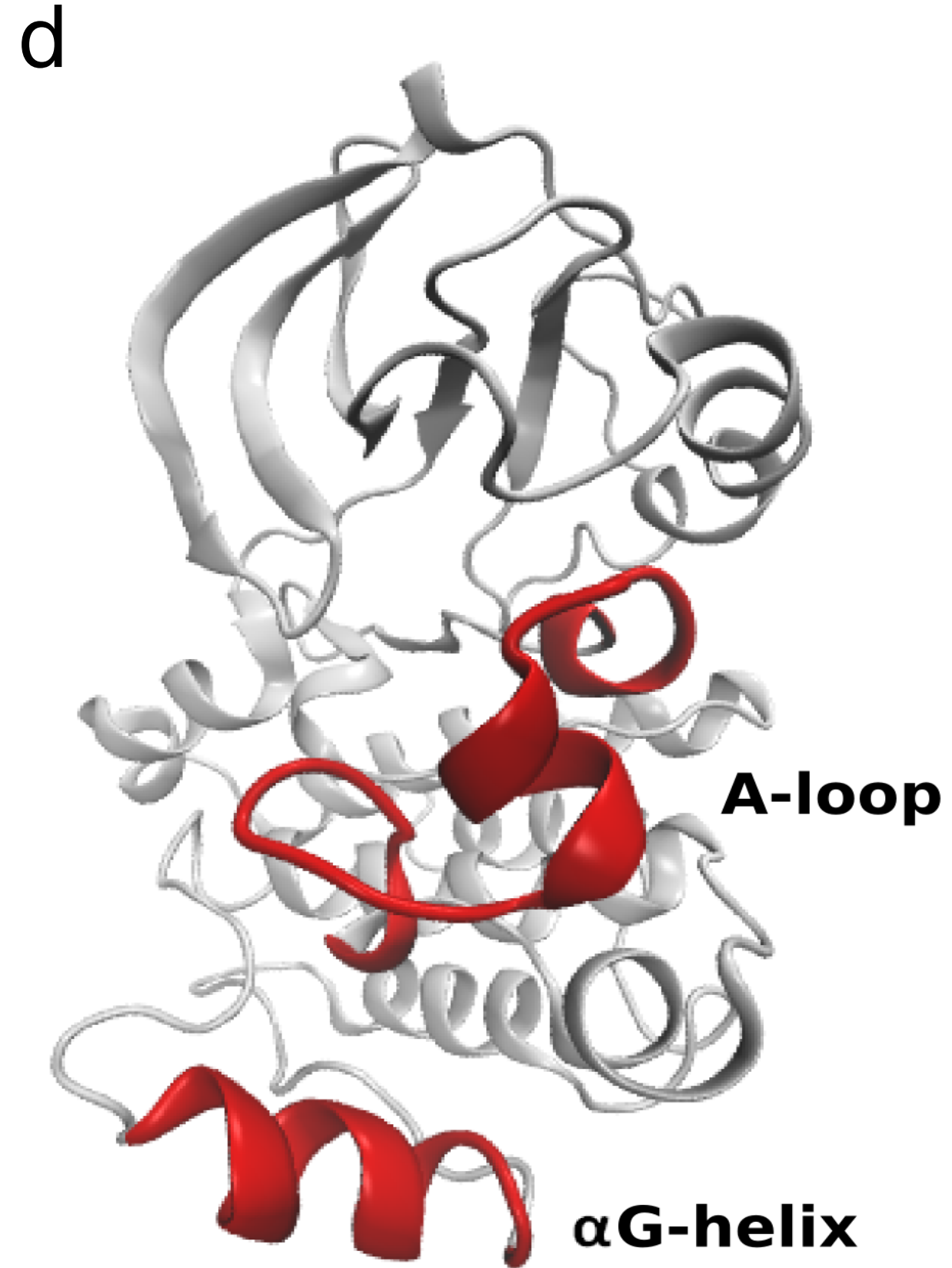

Supplement: S2 Fig — Fluctuations of the αG-helix region for all the Abl drug-resistant mutants (a) and all the tyrosine kinases (b) under study. Shades of red have been used to identify strong imatinib binders, while shades of blue identify weak binders. Solid and dotted lines have been used for clarity. (c,d) Different αG-helix conformations peculiar of the Src kinase. (PDF) [file pcbi.1004578.s002.pdf]

a

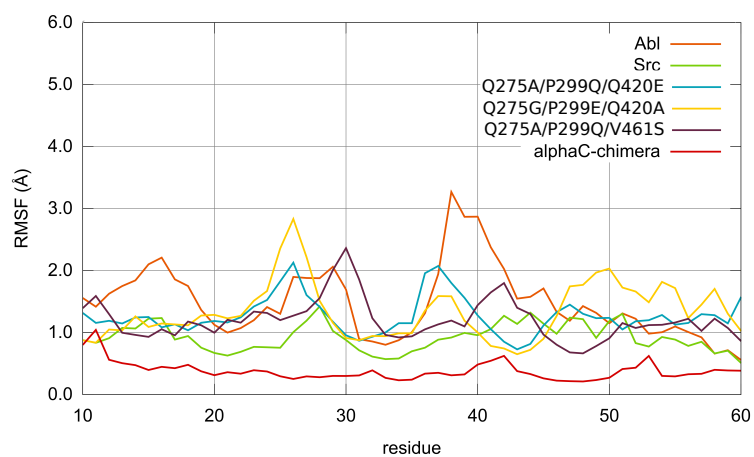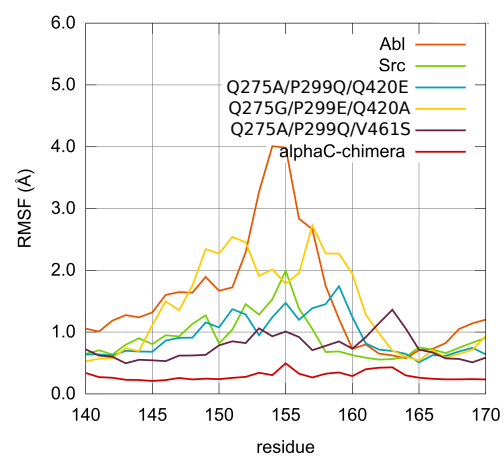

b

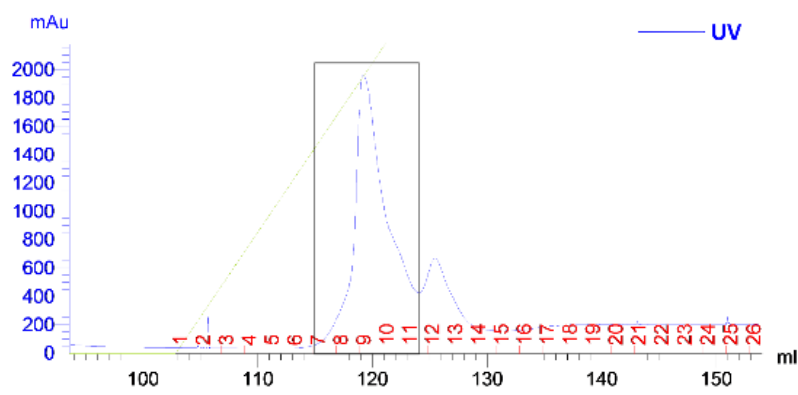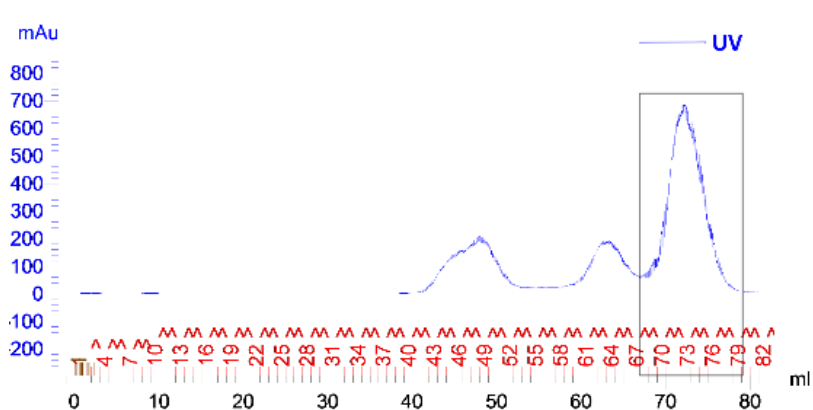

c

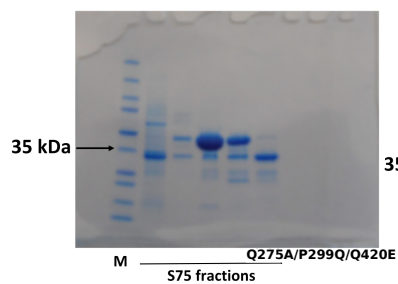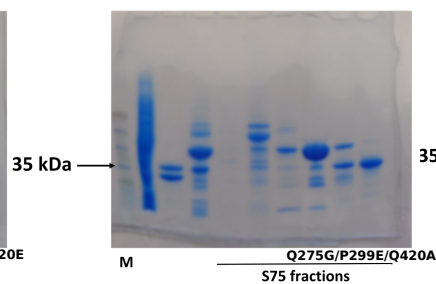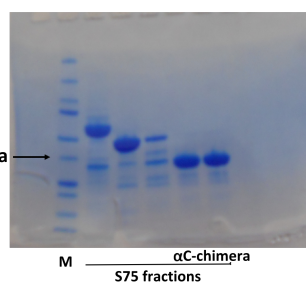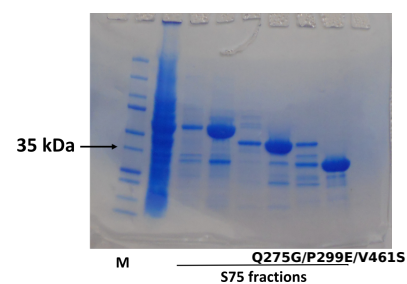

d

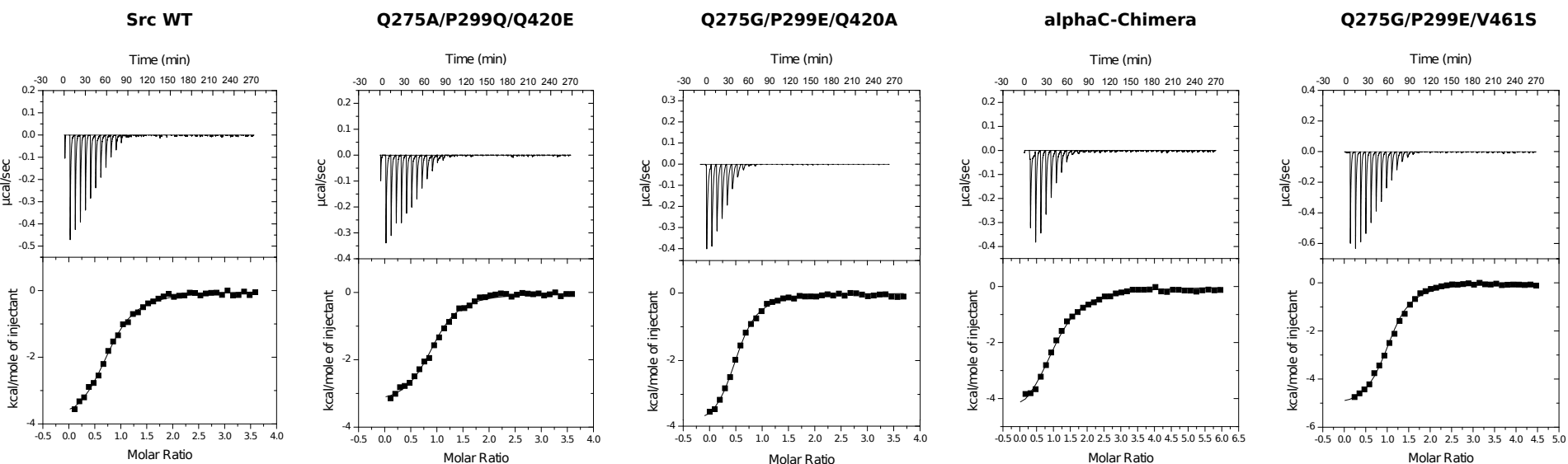

Supplement: S3 Fig — (a) RMSF of the N-lobe (left panel) and the A-loop (right panel) regions for the engineered Src mutants. (b) HisTrap and S75 columns chromatogram; the protein of interest is the more intense UV signal. (c) Gels showing the obtained purified mutants. (d) Titration calorimetry experiments of Src WT and of the designed mutants. (PDF) [file pcbi.1004578.s003.pdf]

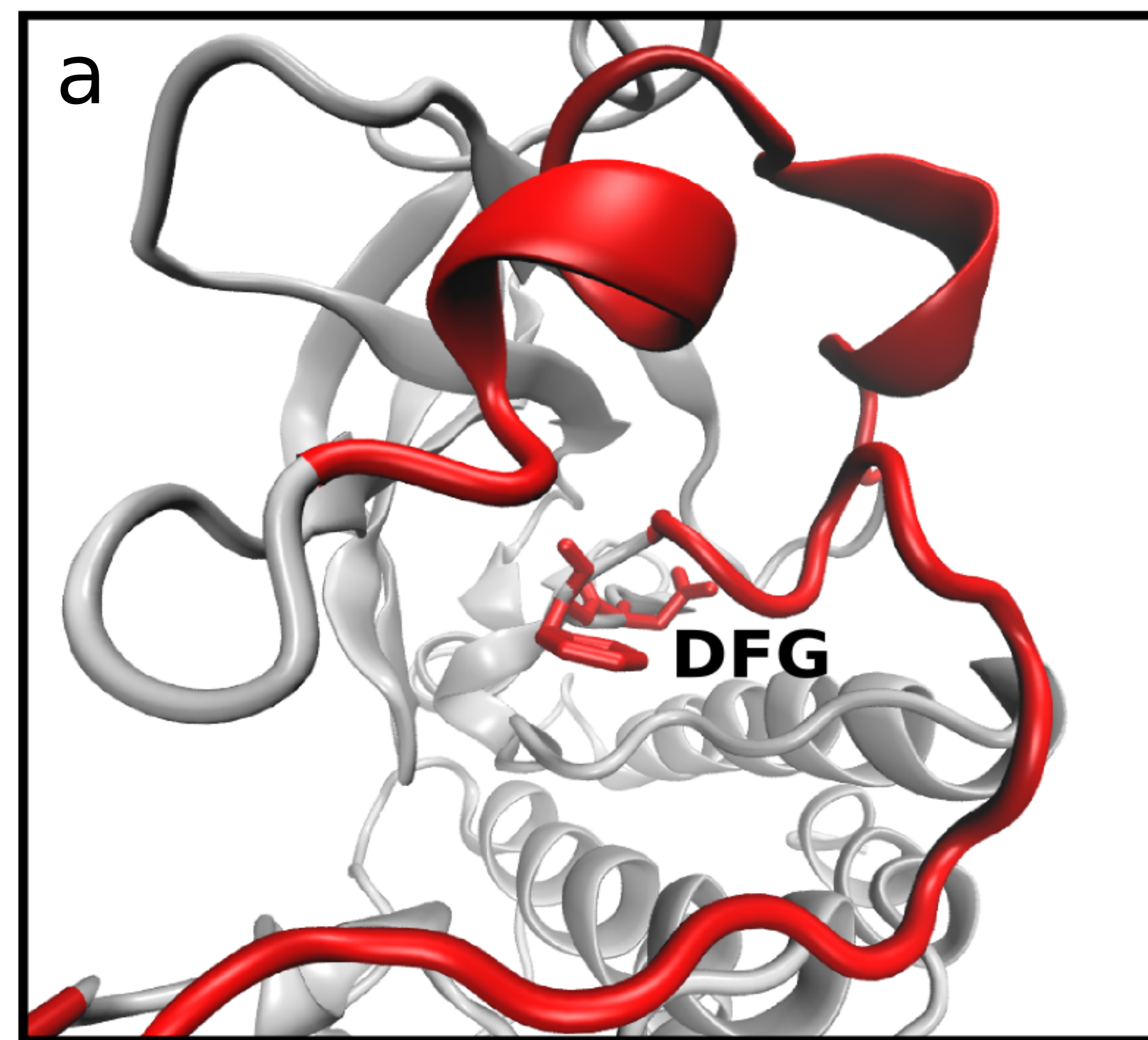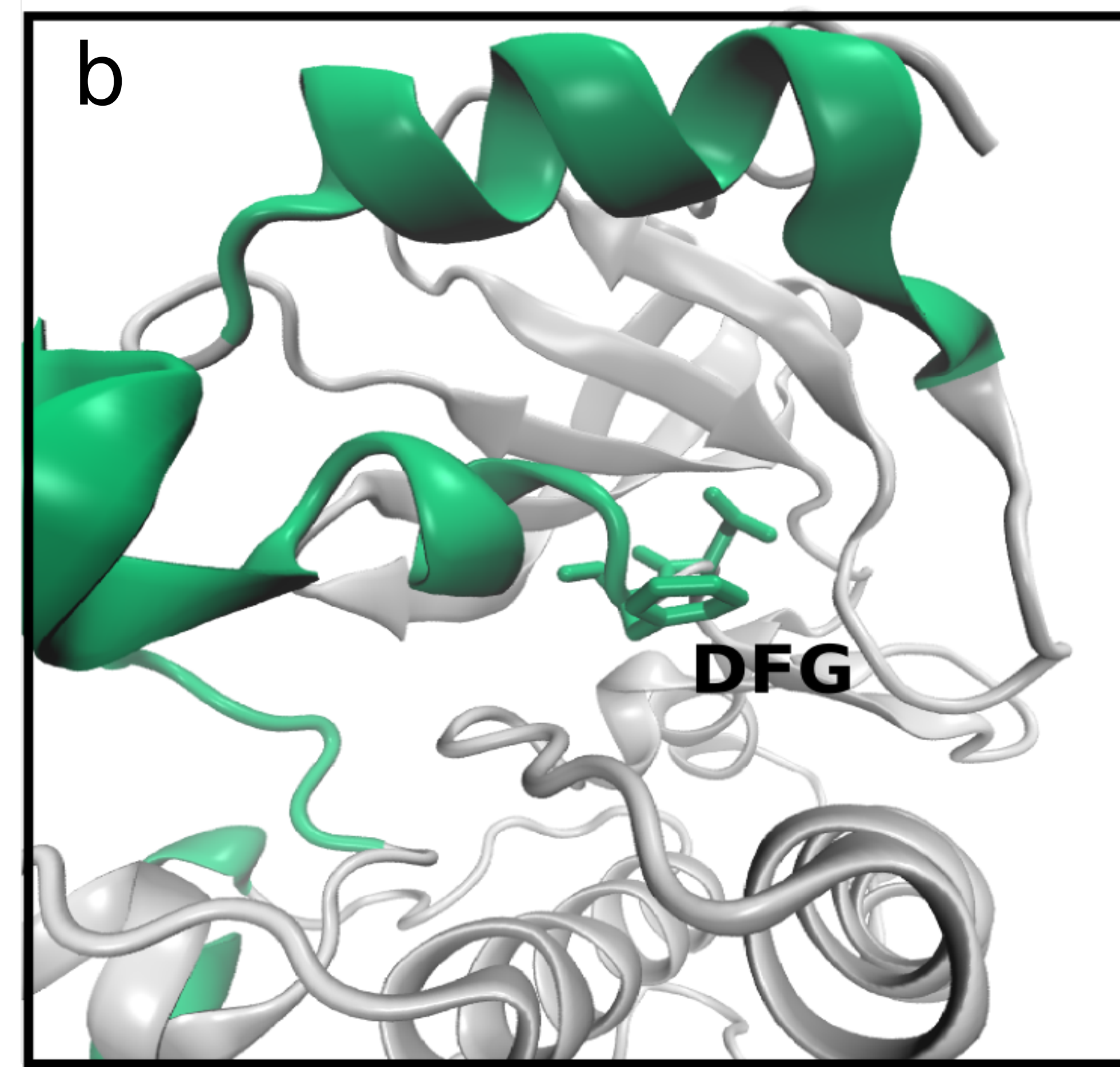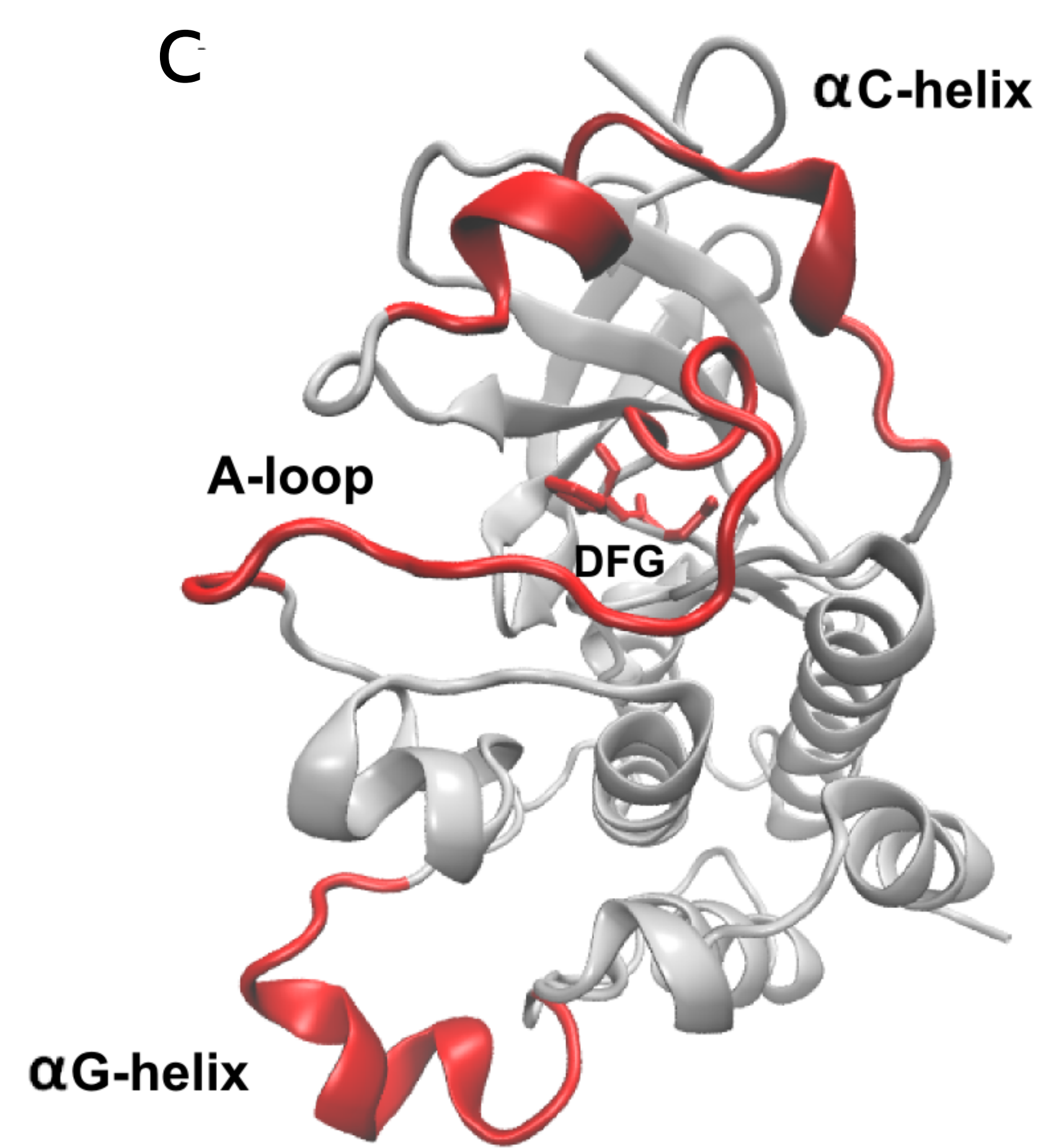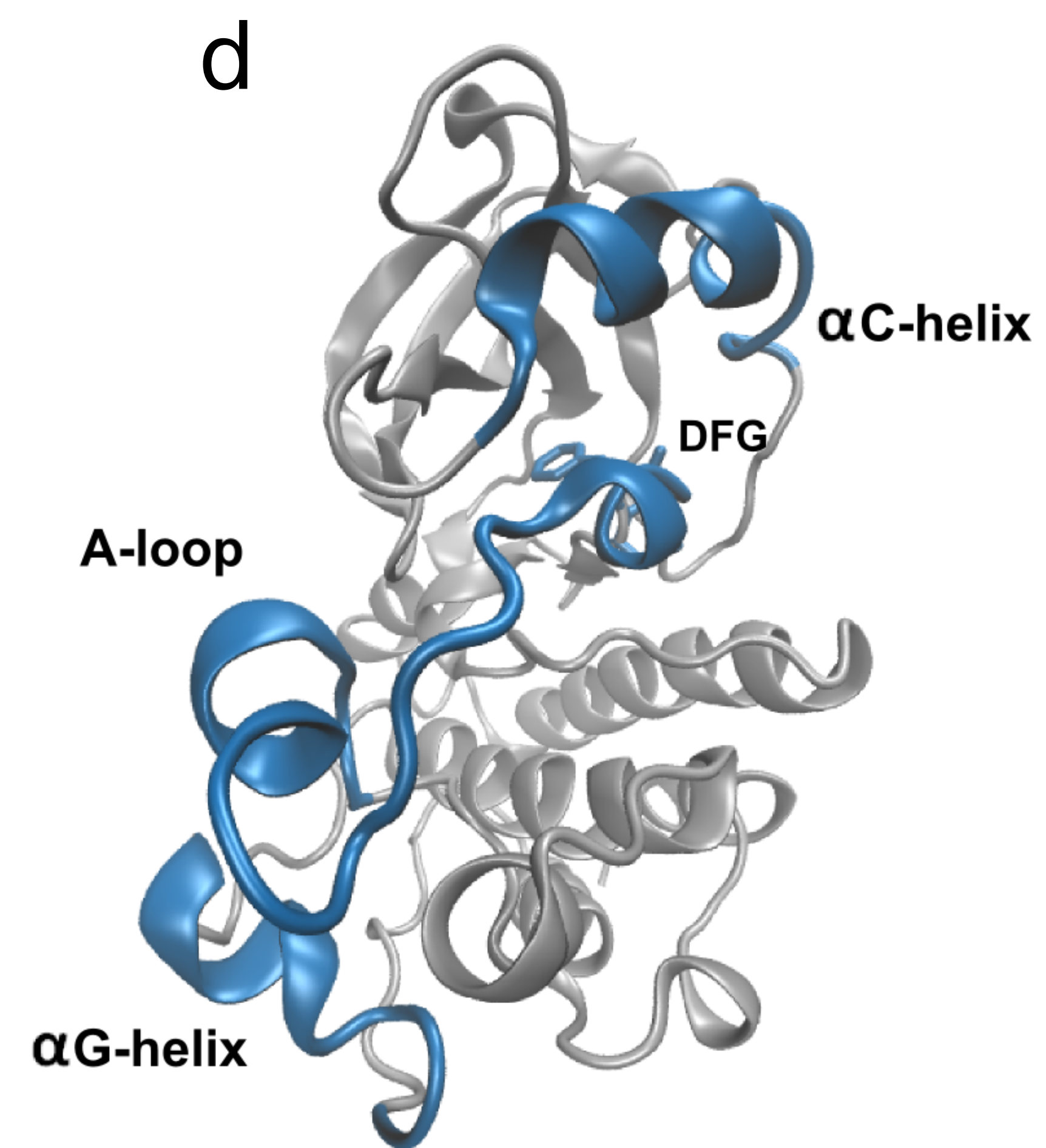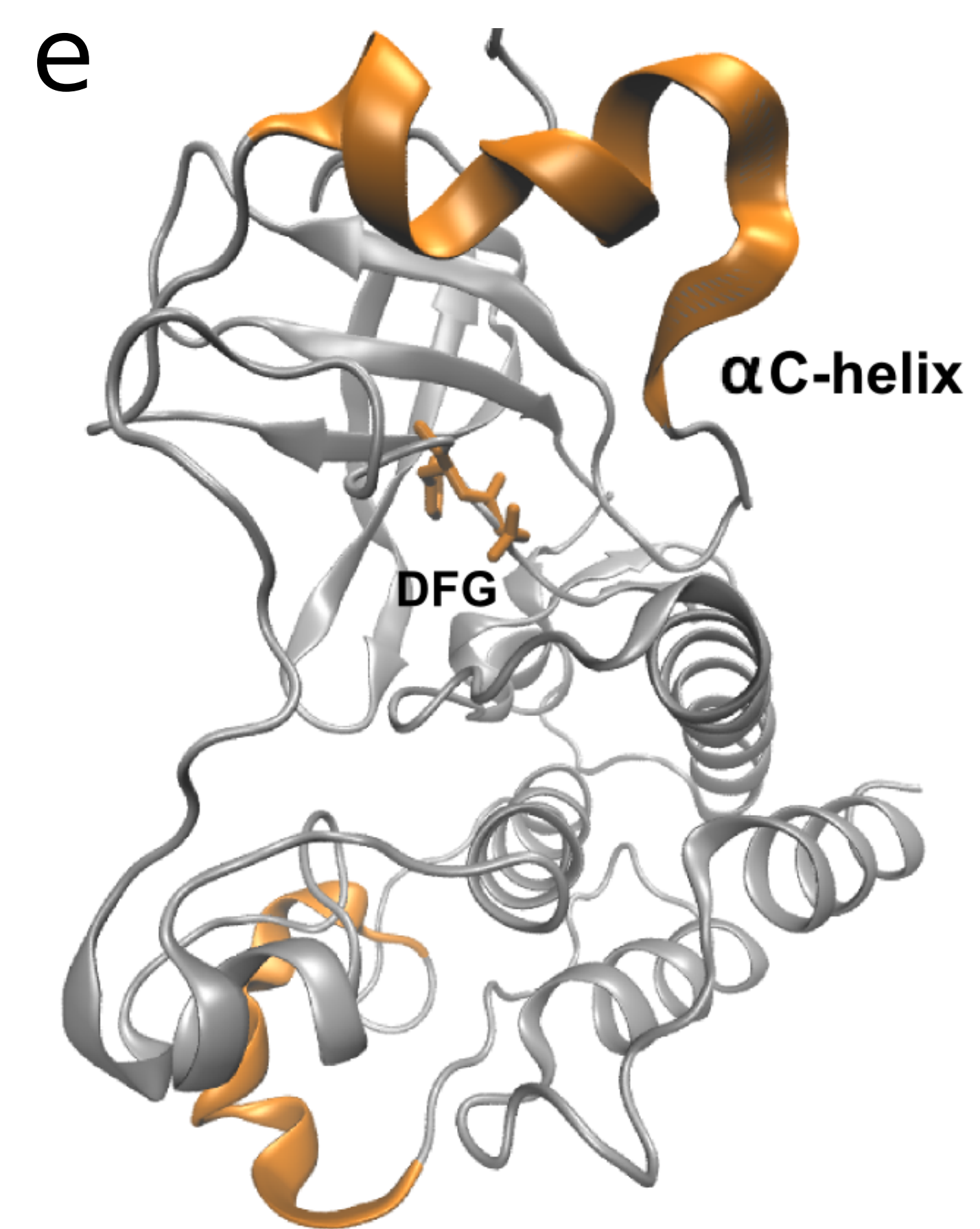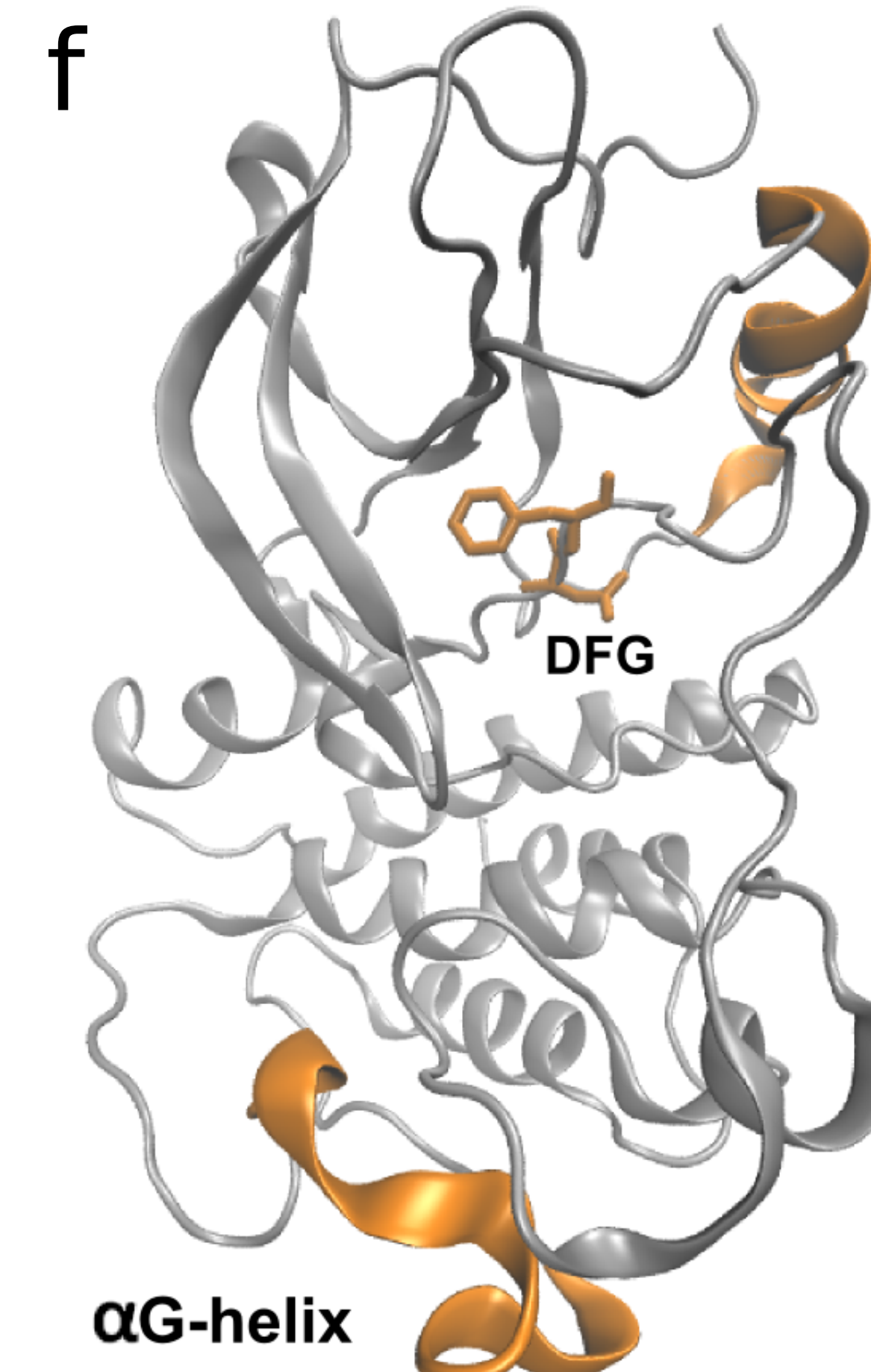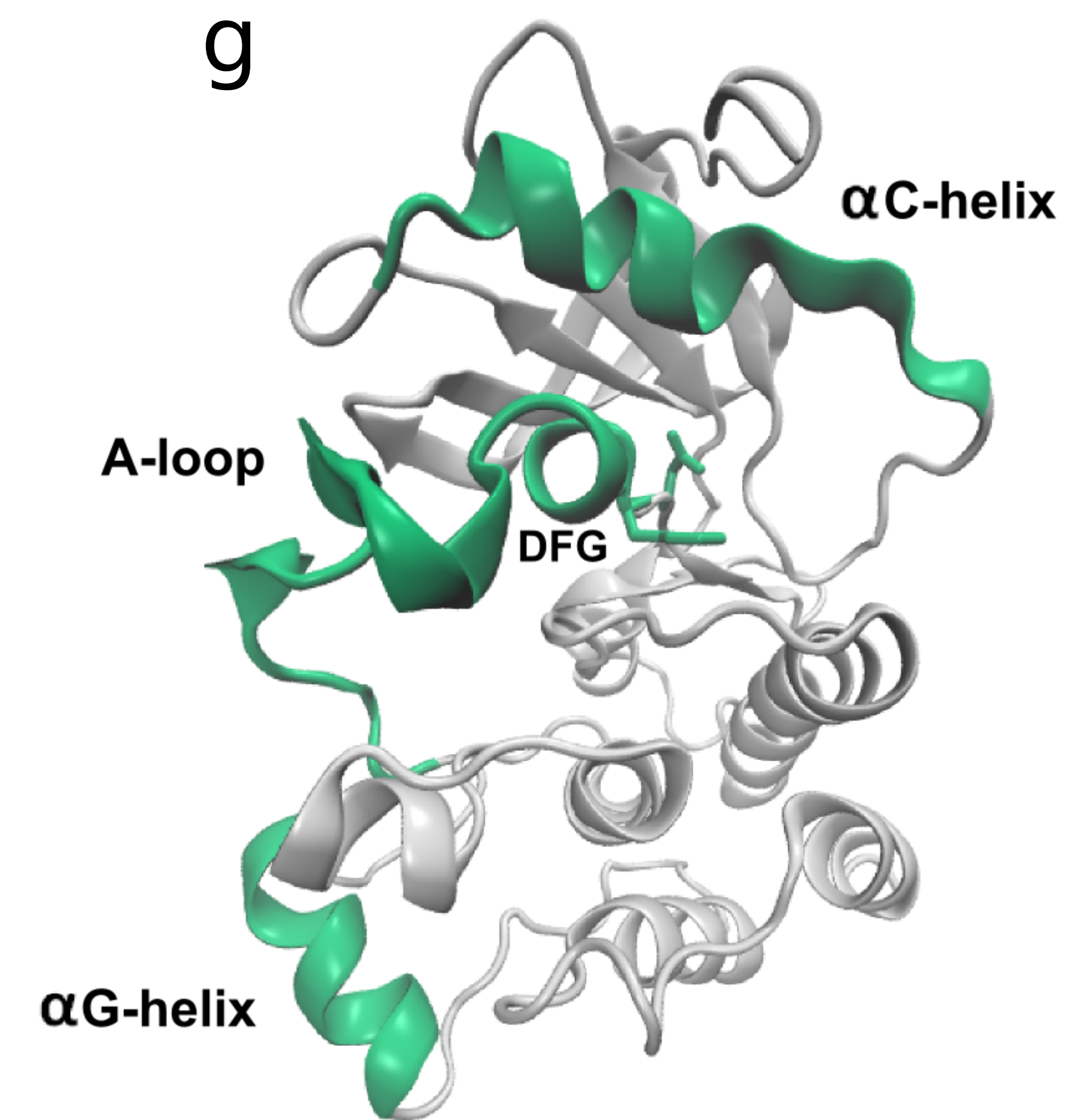

Supplement: S4 Fig — DFG-out state of G250E (a) and E450K (b) with a peculiar out-out geometry. Unfolding event of αC-helix and αG-helix for G250E (c), E279K (d), H396P (e,f) and E450K (g). (PDF) [file pcbi.1004578.s004.pdf]

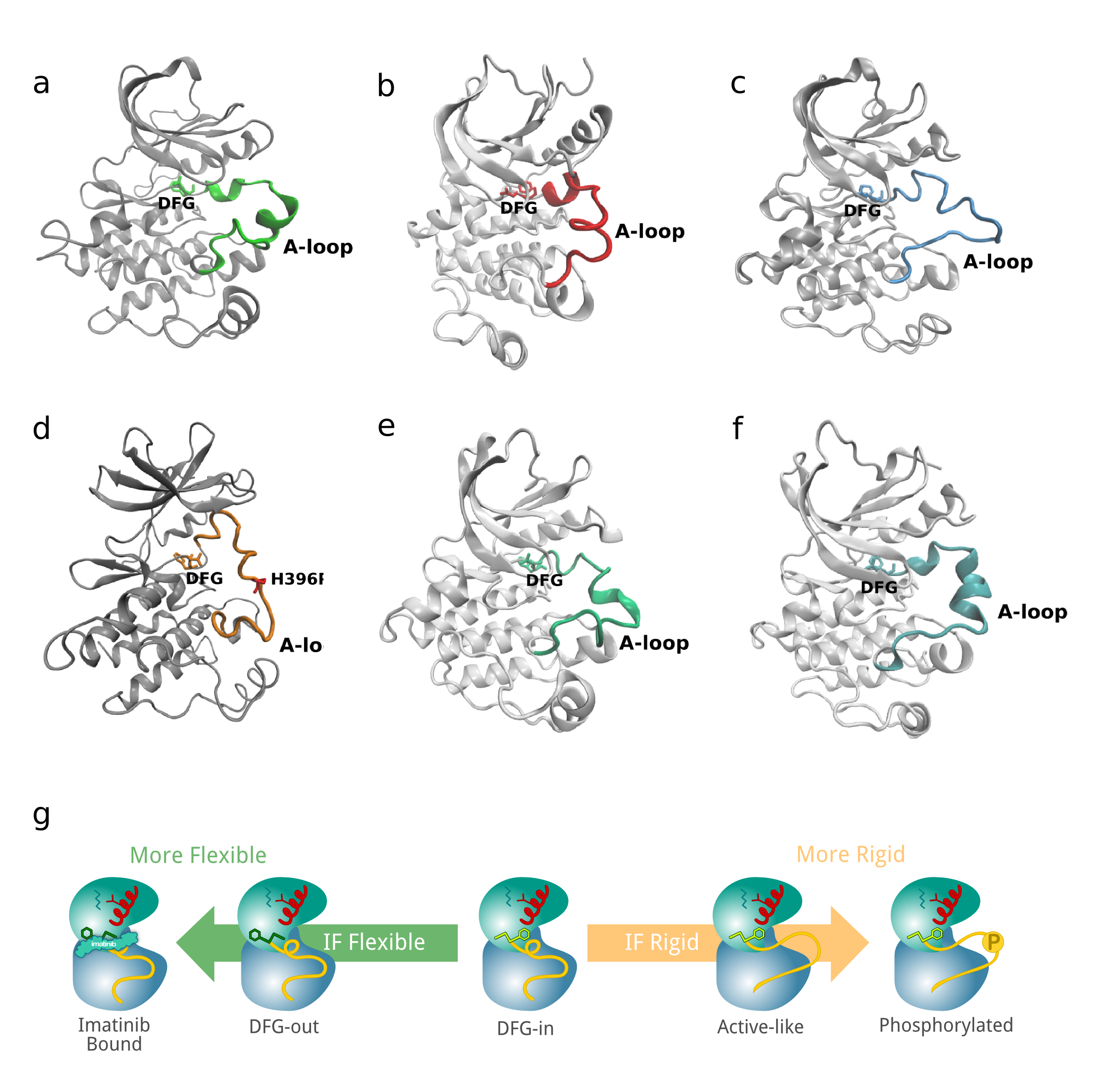

Supplement: S5 Fig — In the mutants the A-loop forms a second helix turn, characteristic of the A-loop of Src, shown in (a). The A-loop conformations of the mutants have been shown as follow: G250E (b), E279K (c), H396P (d), E450K (e) and T315I (f). A schematic representation of the proposed resistance mechanism is shown in (g). The destabilization of the DFG-out inactive state plays a prominent role. (PNG) [file pcbi.1004578.s005.png]

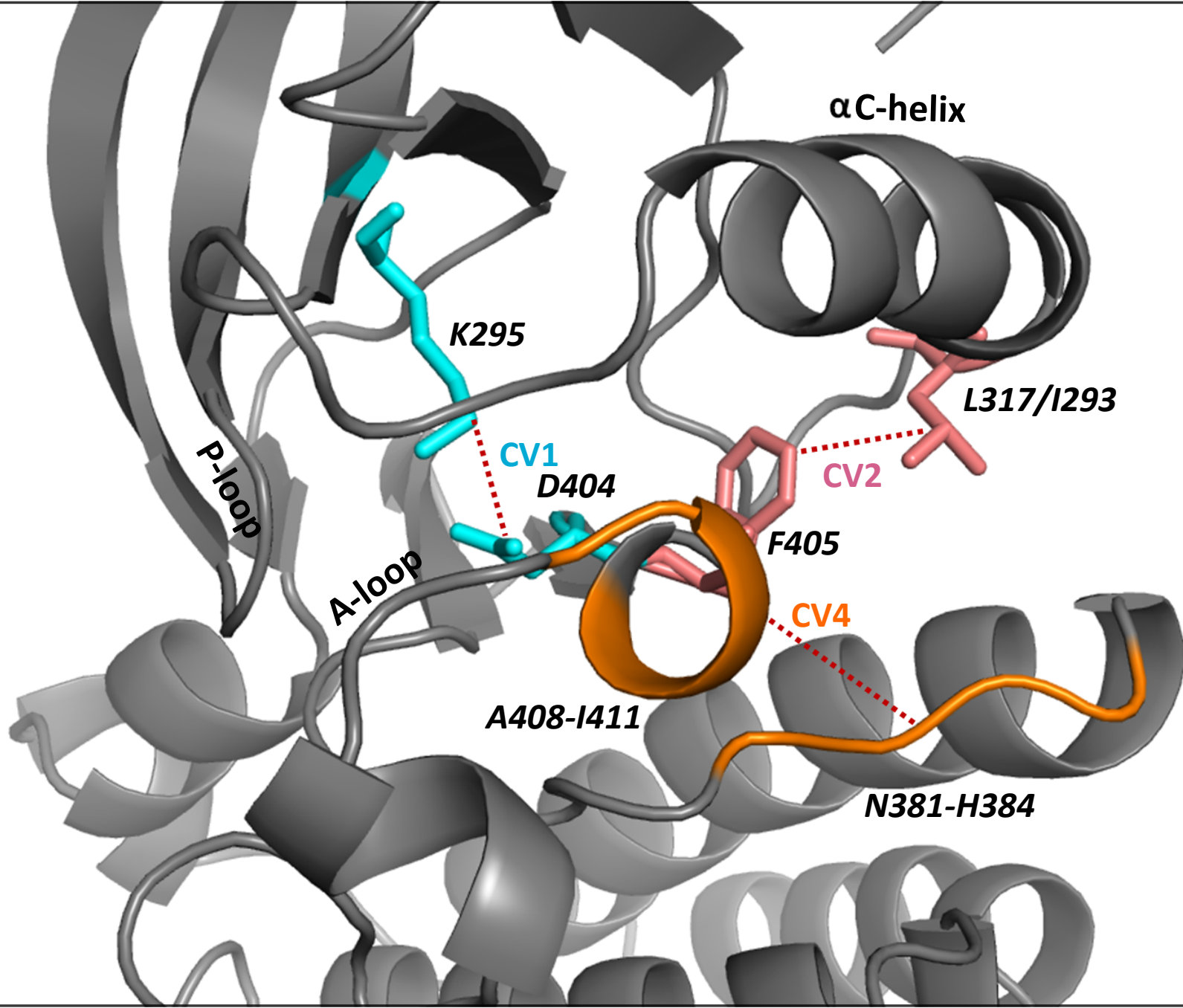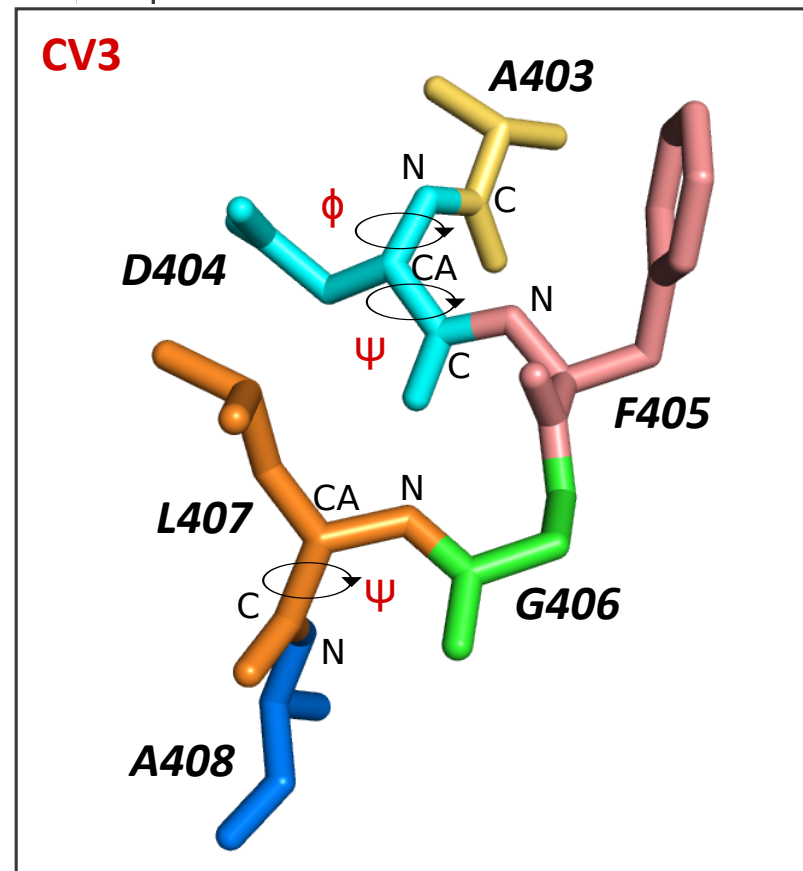

Supplement: S6 Fig — Src numbering is used throughout, with the exception of residue L317, that corresponds to residue I293 in Abl (PDB:G1T numbering used). The dihedral angle combination f(ϕ 404, ψ 405, ψ 408) (CV3) is shown on the right. (PDF) [file pcbi.1004578.s006.pdf]

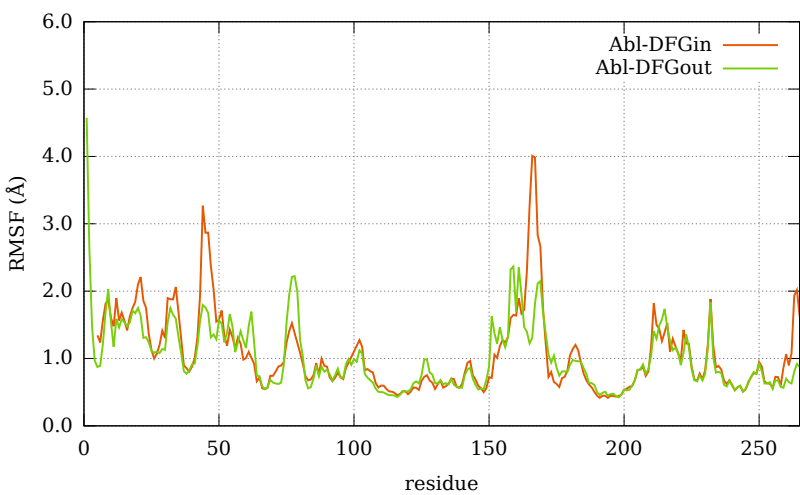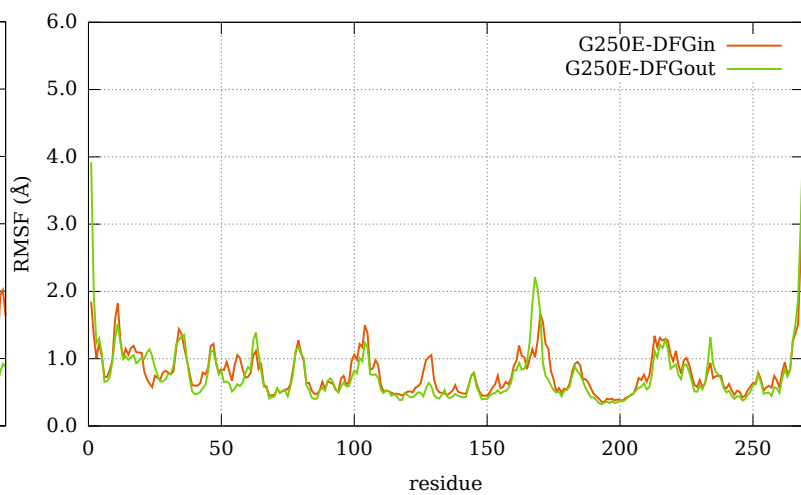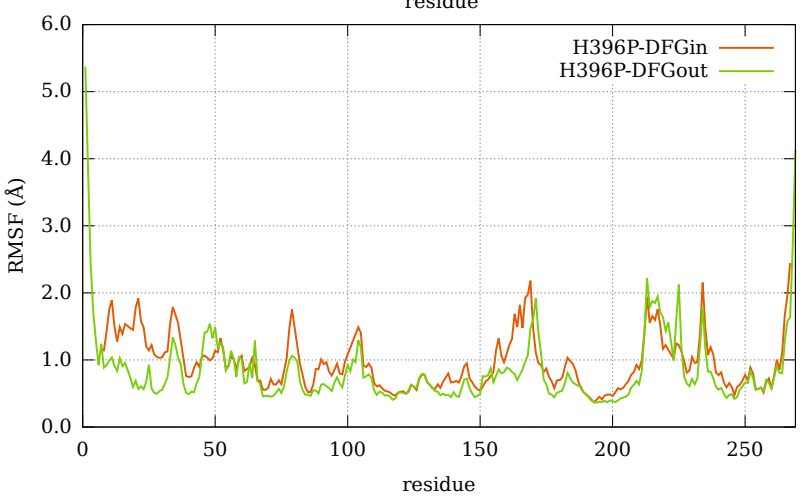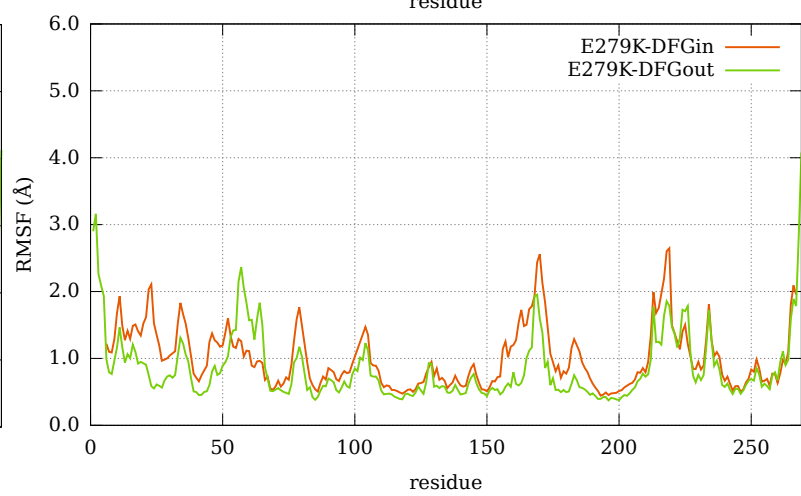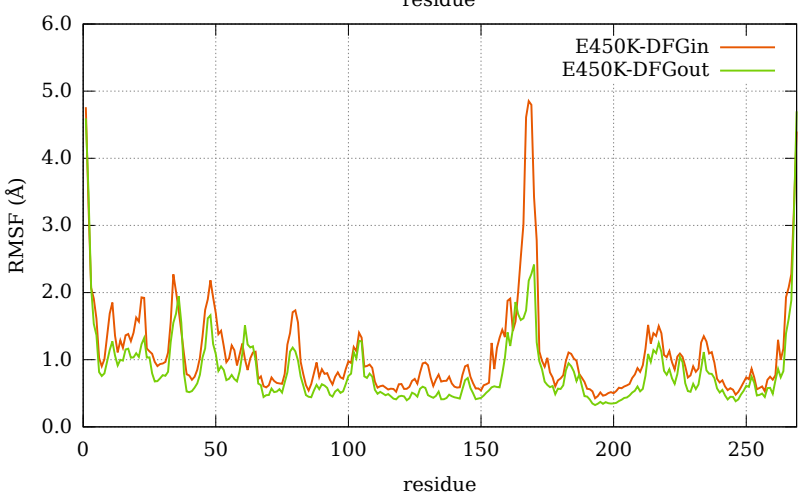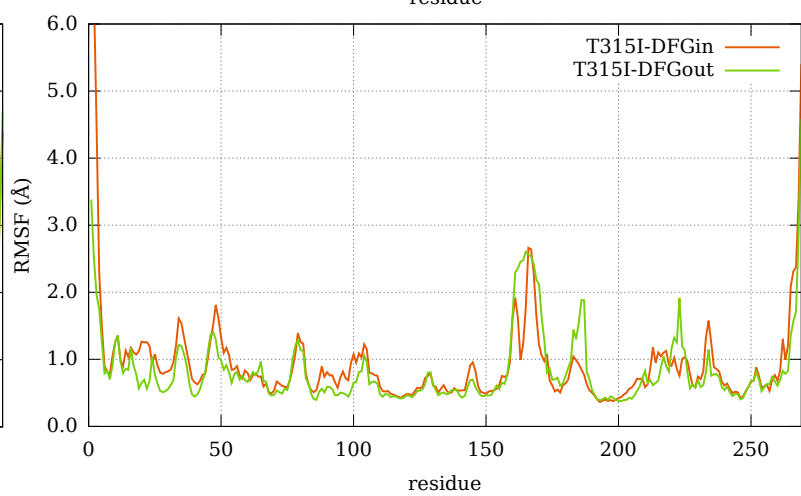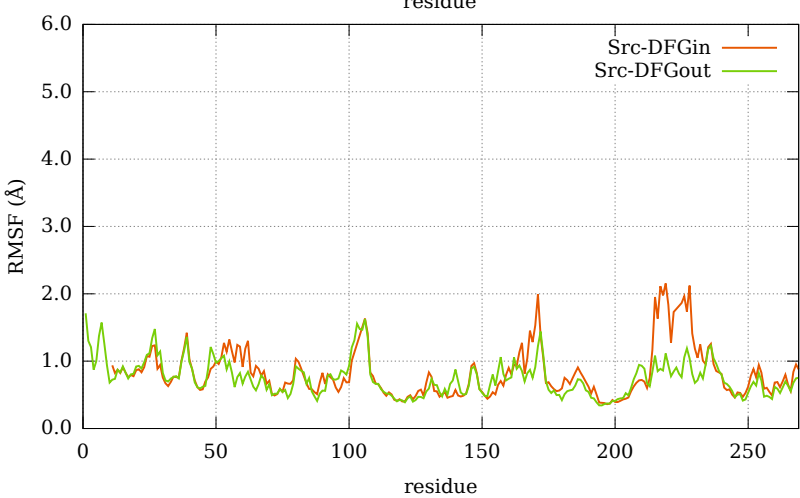

Supplement: S7 Fig — RMSF profiles of DFG-in (in orange) and DFG-out (in green) states for Src, Abl and the resistant mutants of Abl. (PDF) [file pcbi.1004578.s007.pdf]

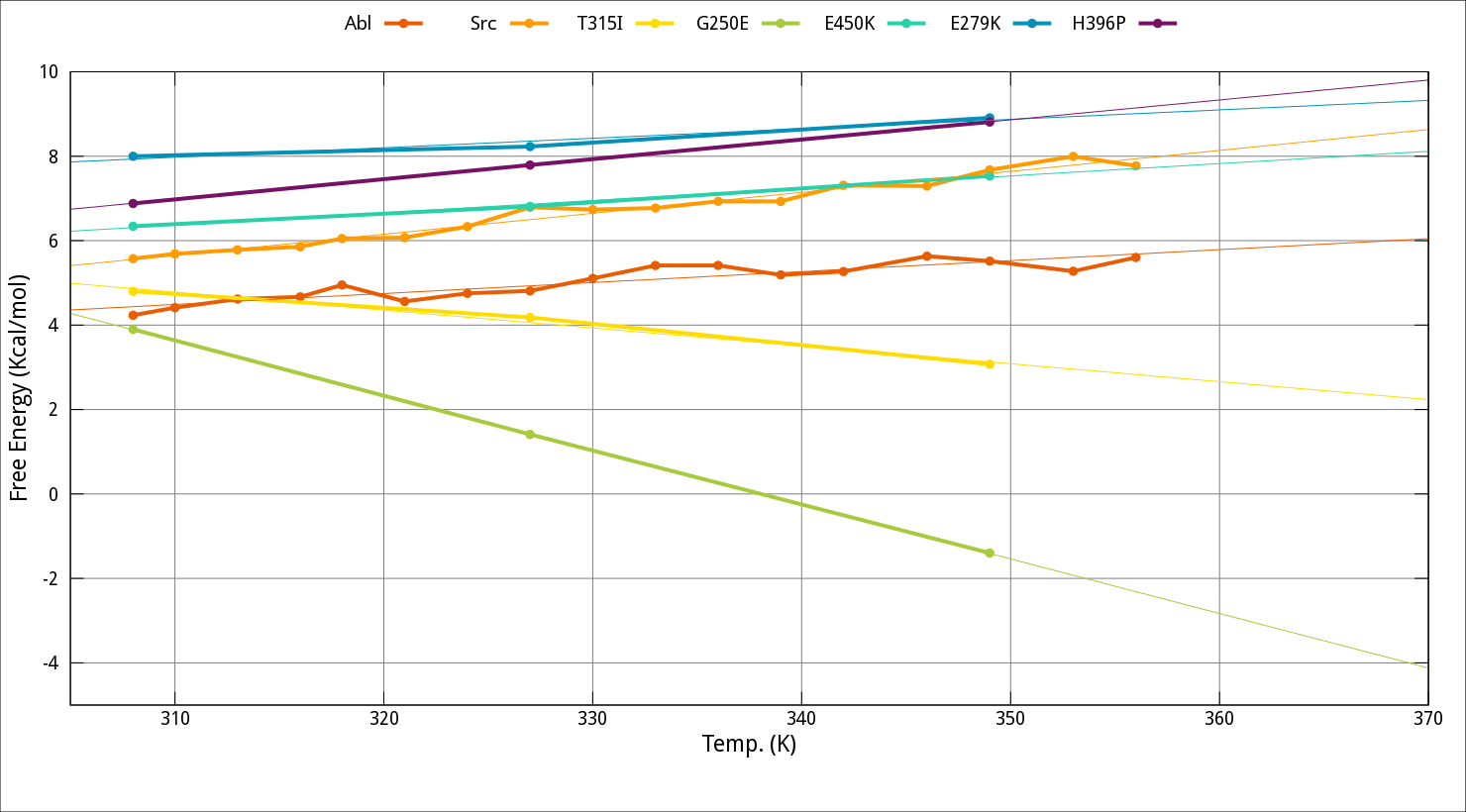

Supplement: S8 Fig — Linear regressions of the PTmetaD free energies as a function of temperature. Entropy and Enthalpy contributions have been obtained for Src, Abl and all the resistant mutants. (PNG) [file pcbi.1004578.s008.png]

# Abl

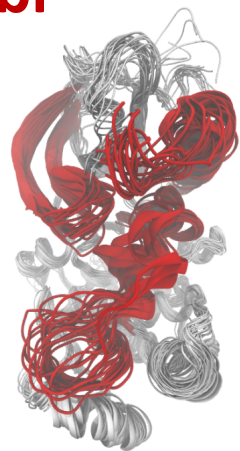

## G250E

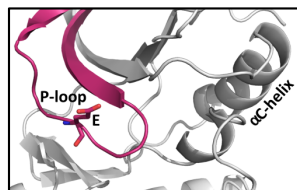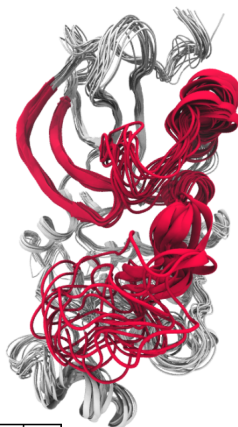

## E279K

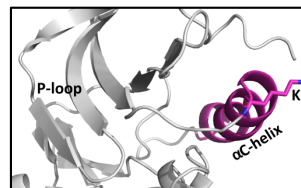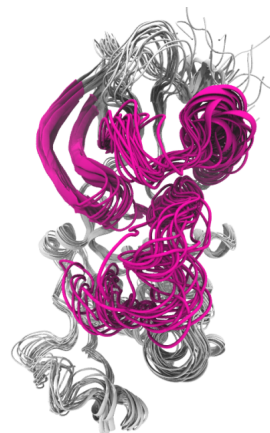

## H396P

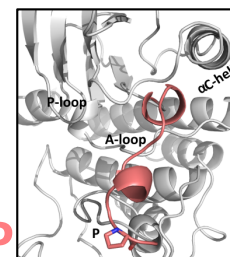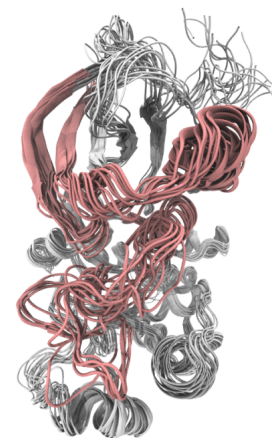

# Src

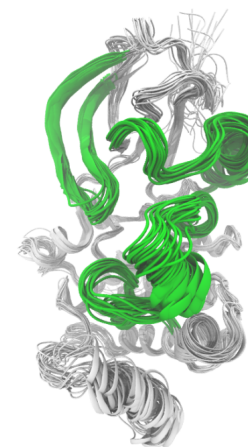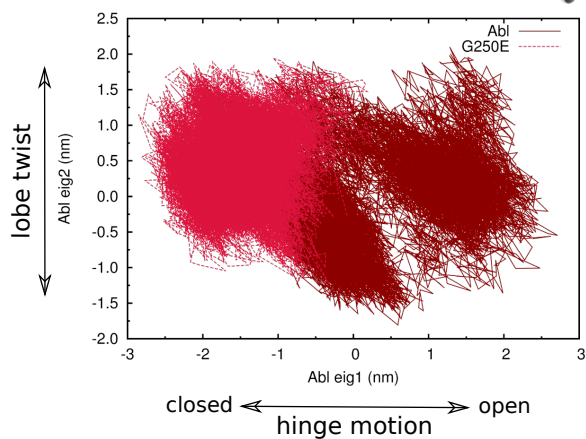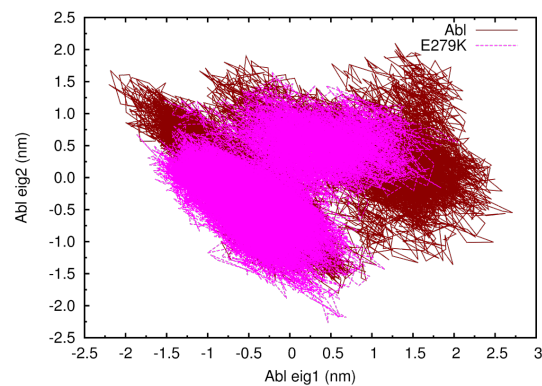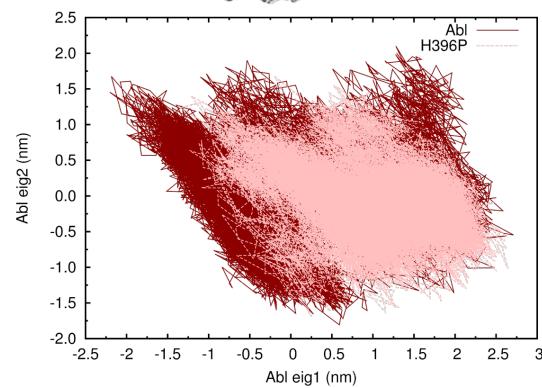

Supplement: S9 Fig — PCA analysis for Abl, Src and the resistant mutants of Abl. The trajectories of Src and of the resistant mutants of Abl have been projected along the first (hinge motion) and second (lobe twist) eigenvector of Abl. (PDF) [file pcbi.1004578.s009.pdf]

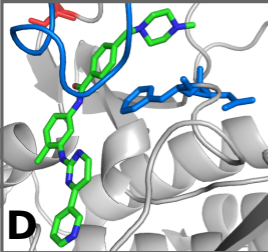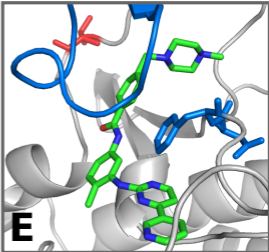

Supplement: S10 Fig — States D and E corresponding to the alternative unbinding path of imatinib in the T315I mutant shown in Fig 5 of the main text. (PDF) [file pcbi.1004578.s010.pdf]

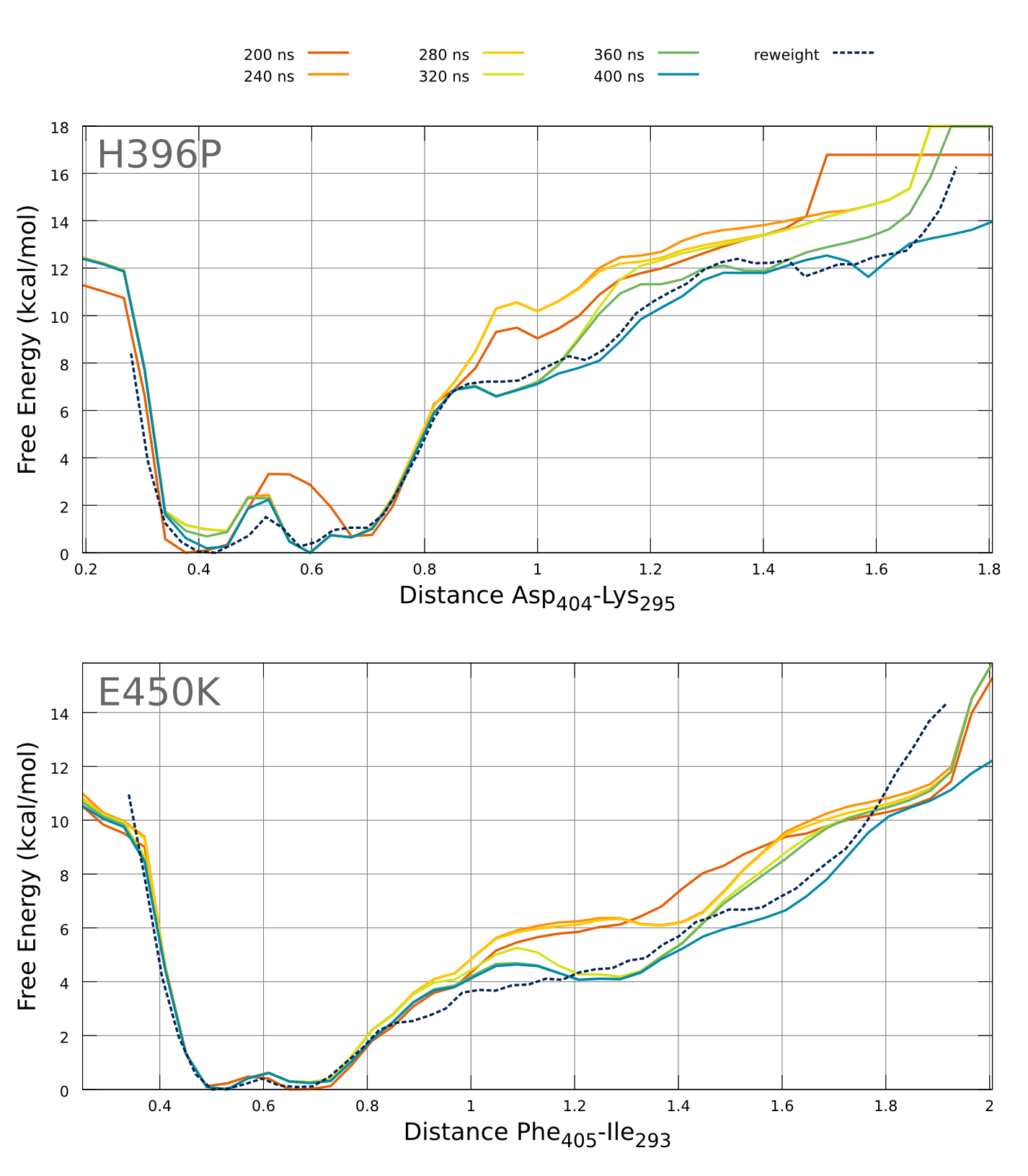

Supplement: S11 Fig — Typical convergence of the PTmetaD calculations. Projection of the free-energy of H396P along CV1 (distance Asp404-Lys295) and of E450K along CV2 (distance Phe405-Ile293), calculated on the final 200 ns of run, at intervals of 40 ns. The dotted lines correspond to the reweight performed on both simulations. (PNG) [file pcbi.1004578.s011.png]

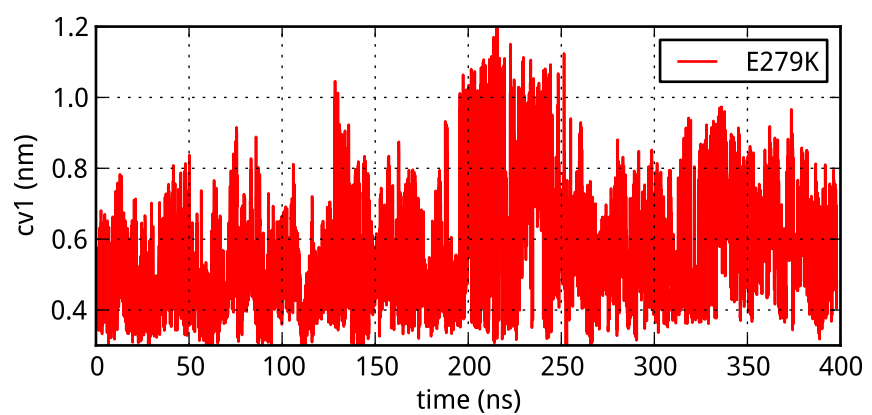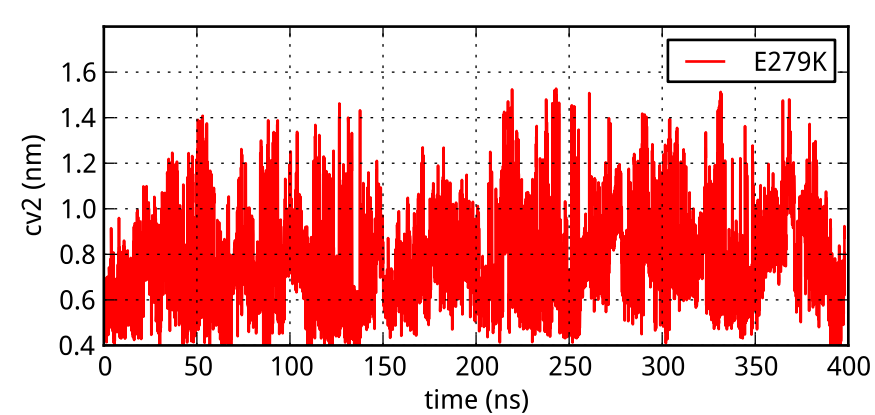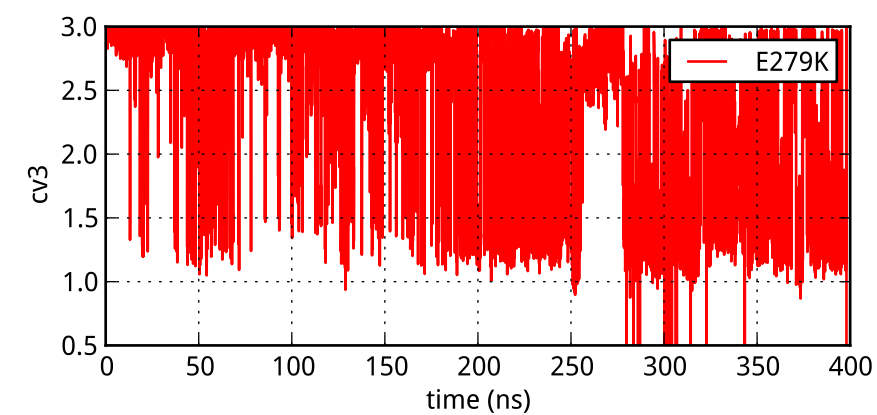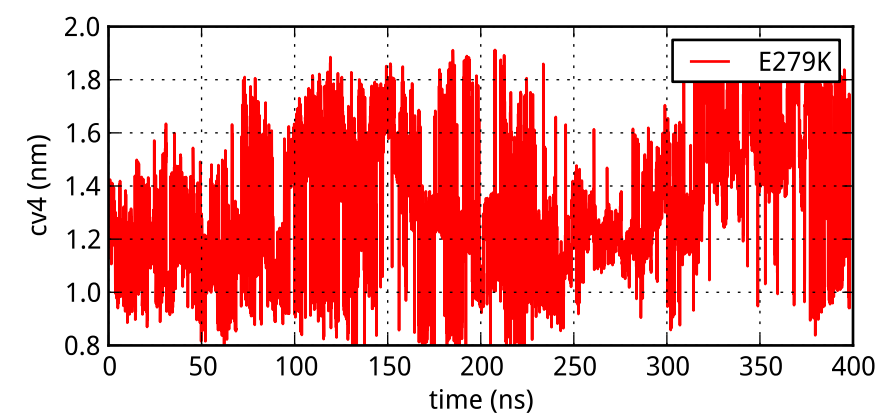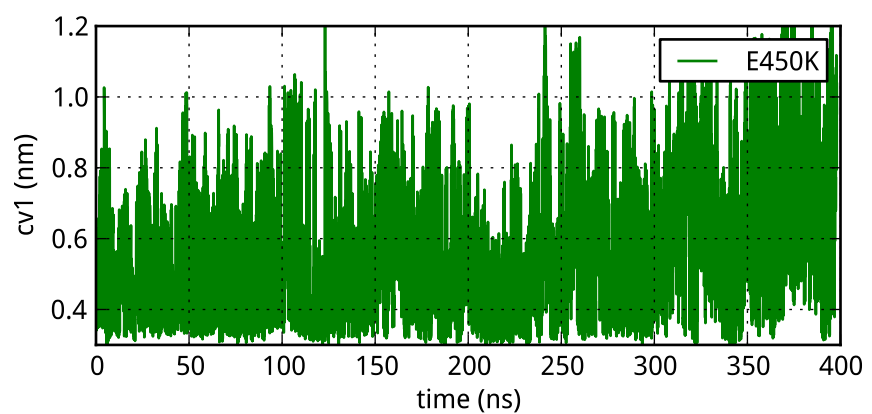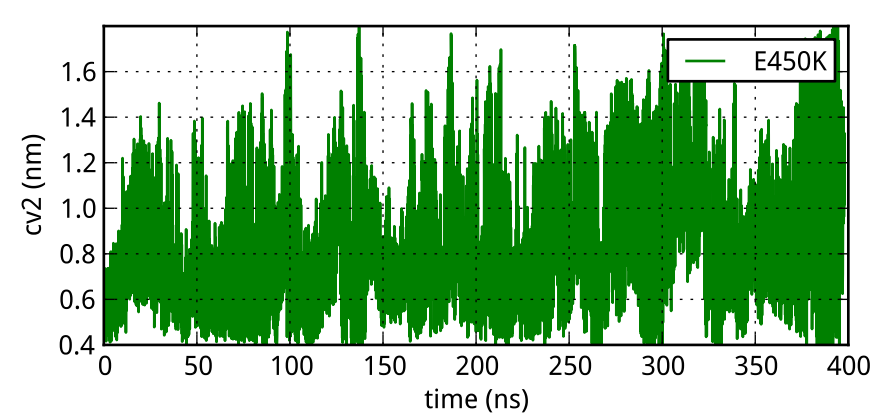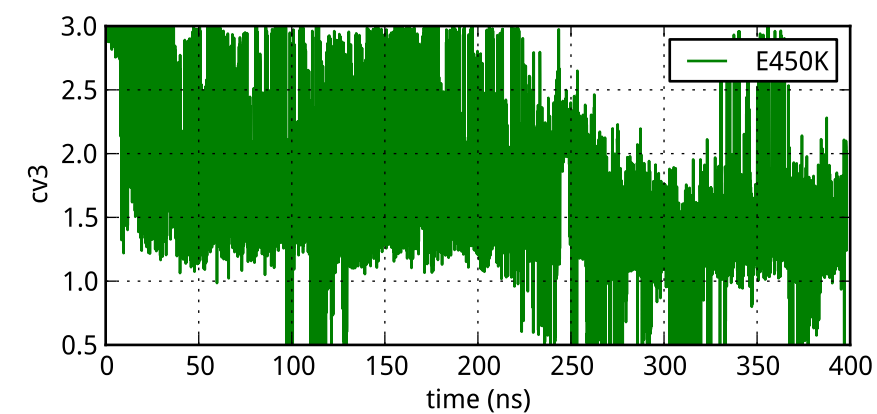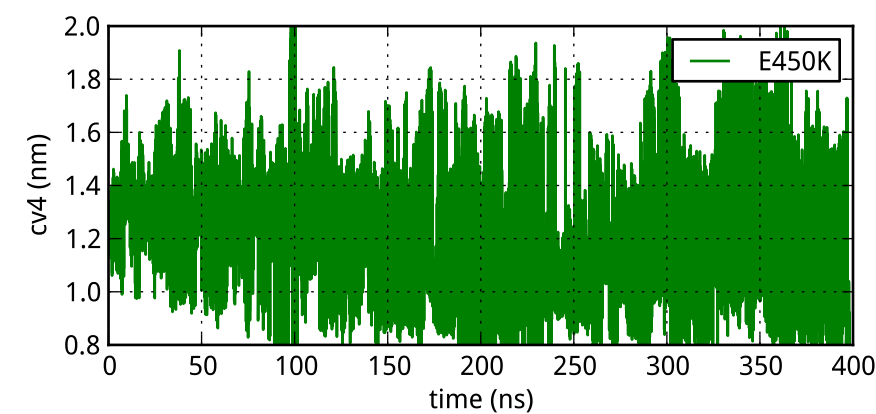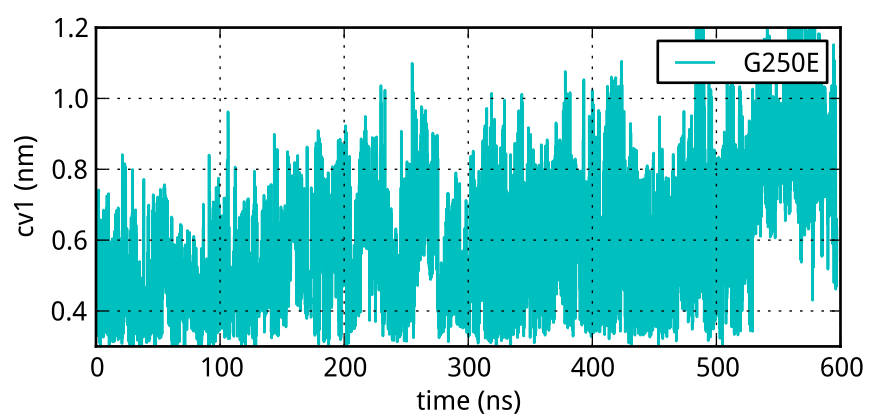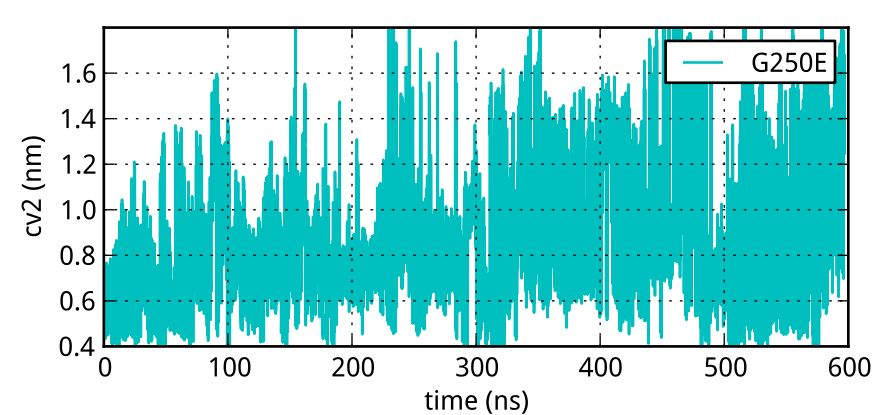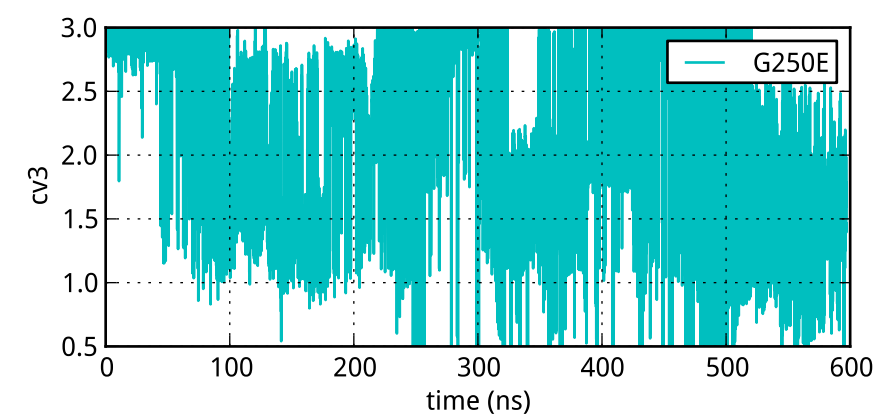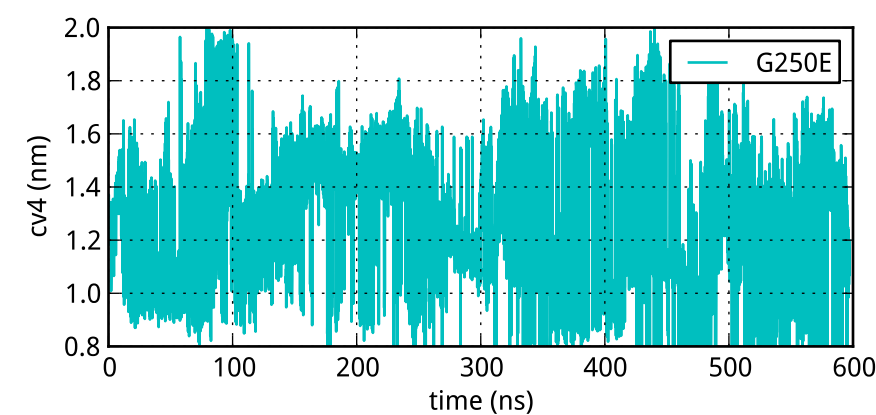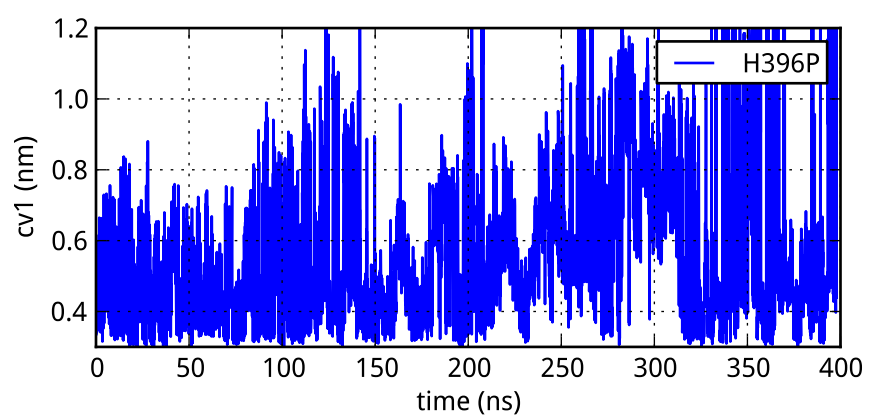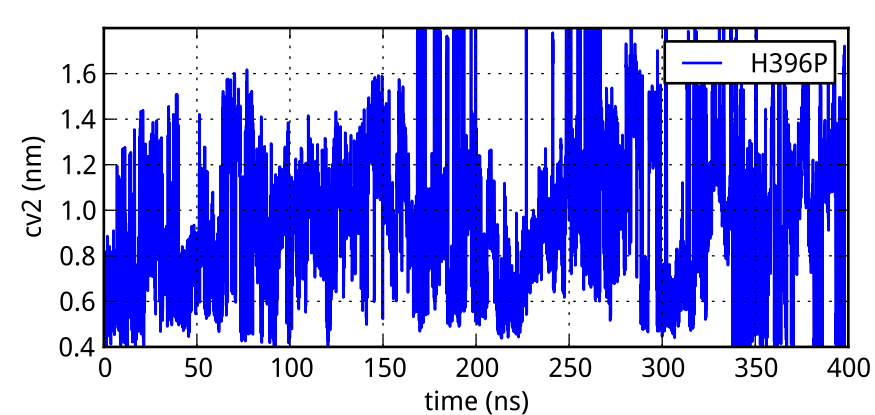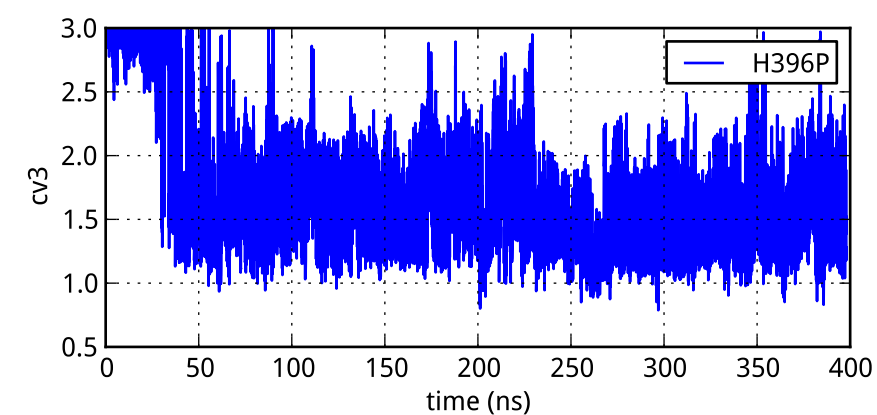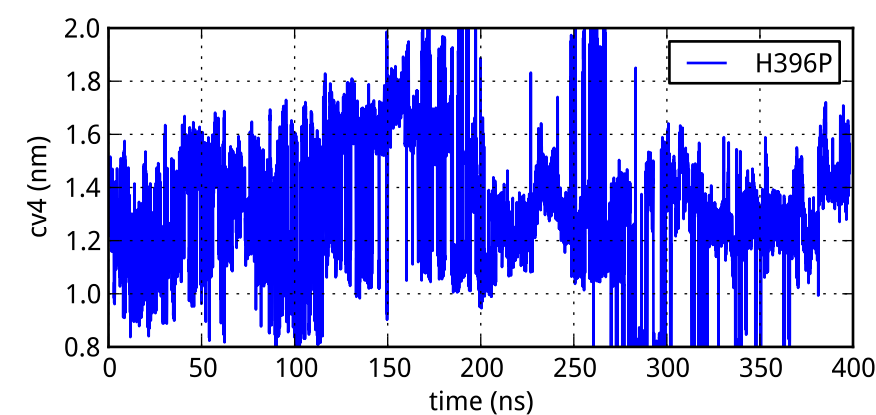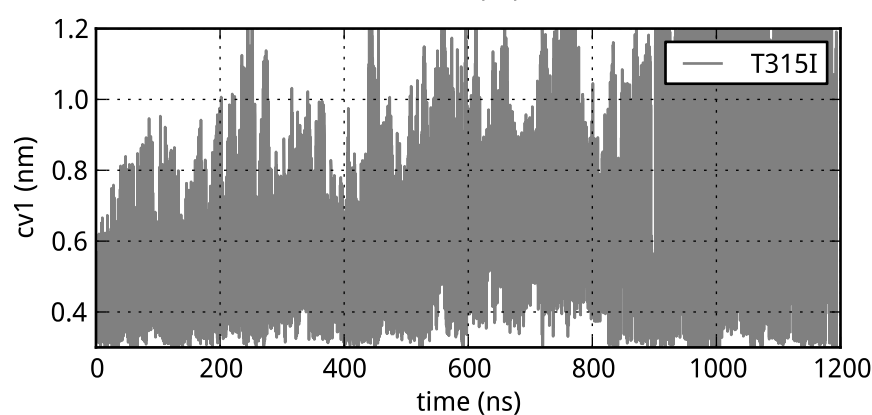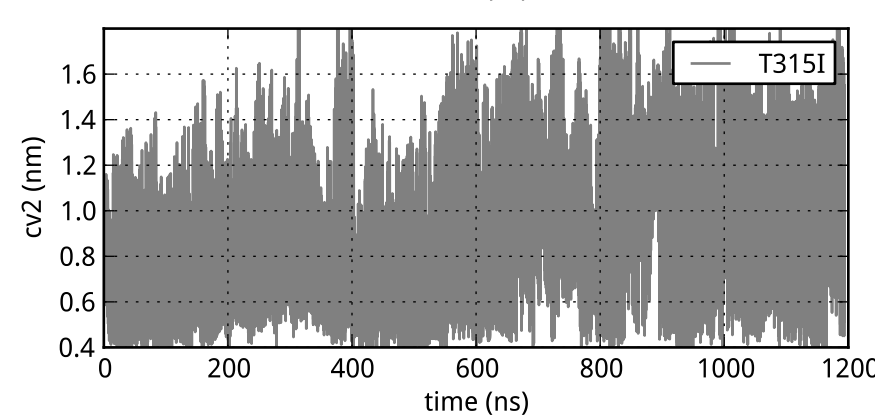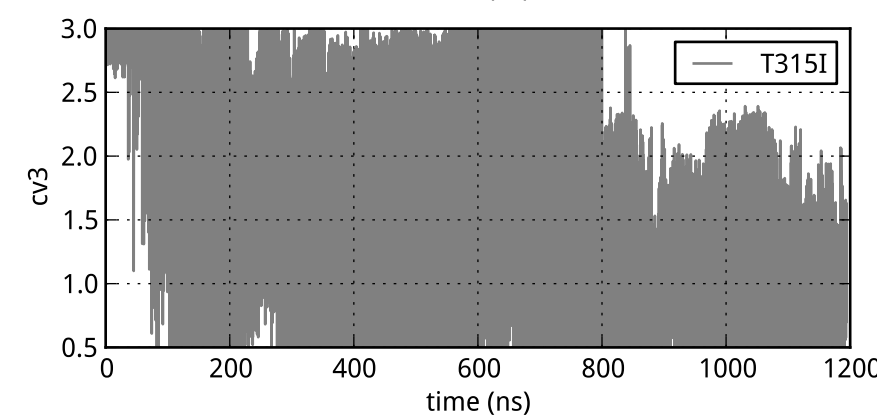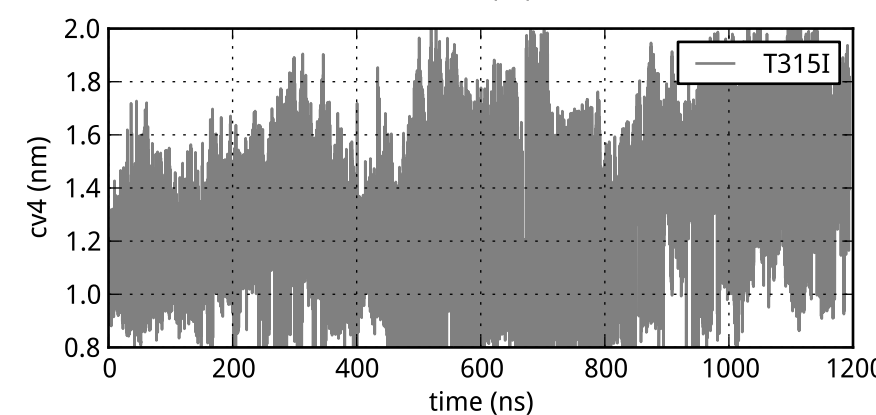

Supplement: S12 Fig — Values of the CVs used for the PTMetaD versus simulation time. In accordance with the behaviour expected, the system diffuses freely in the CV space and visits all the basins within the threshold energy multiple times. (PDF) [file pcbi.1004578.s012.pdf]

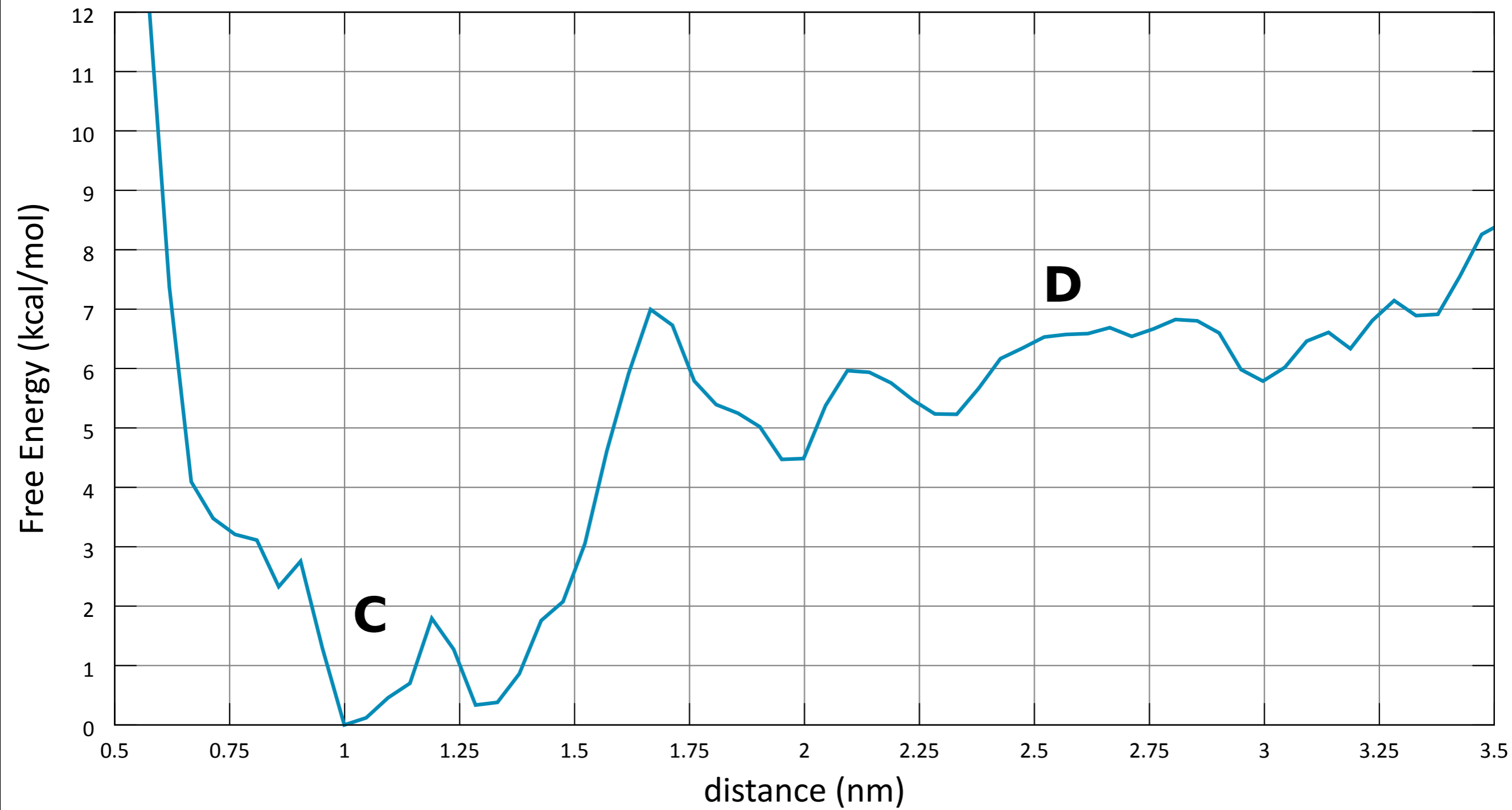

Supplement: S13 Fig — Monodimensional profile of unbinding for Abl WT along the distance between the center of mass of residues Asp404-Leu322-Val323 and Imatinib. The state C corresponds to the external binding pose of Imatinib, while in state D imatinib is unbound and fully solvated. (PDF) [file pcbi.1004578.s013.pdf]

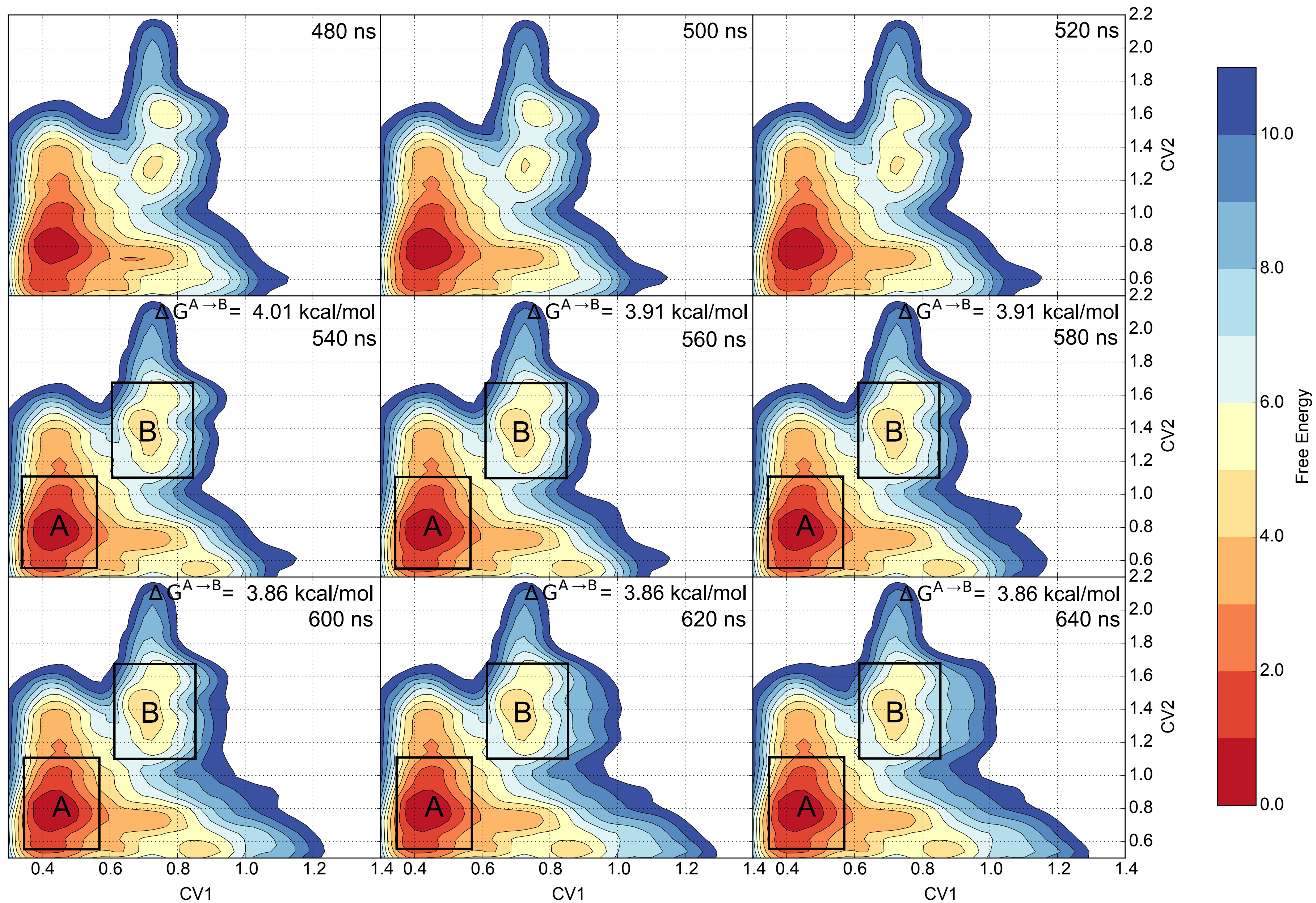

Supplement: S14 Fig — Bidimensional profiles of G250E along CV1 and CV2 calculated at intervals of 20 ns in the last 160 ns of simulation time. The very small changes in the FES and the consistency of the relative ΔGs gives a good indication of the sampling convergence. (PDF) [file pcbi.1004578.s014.pdf]

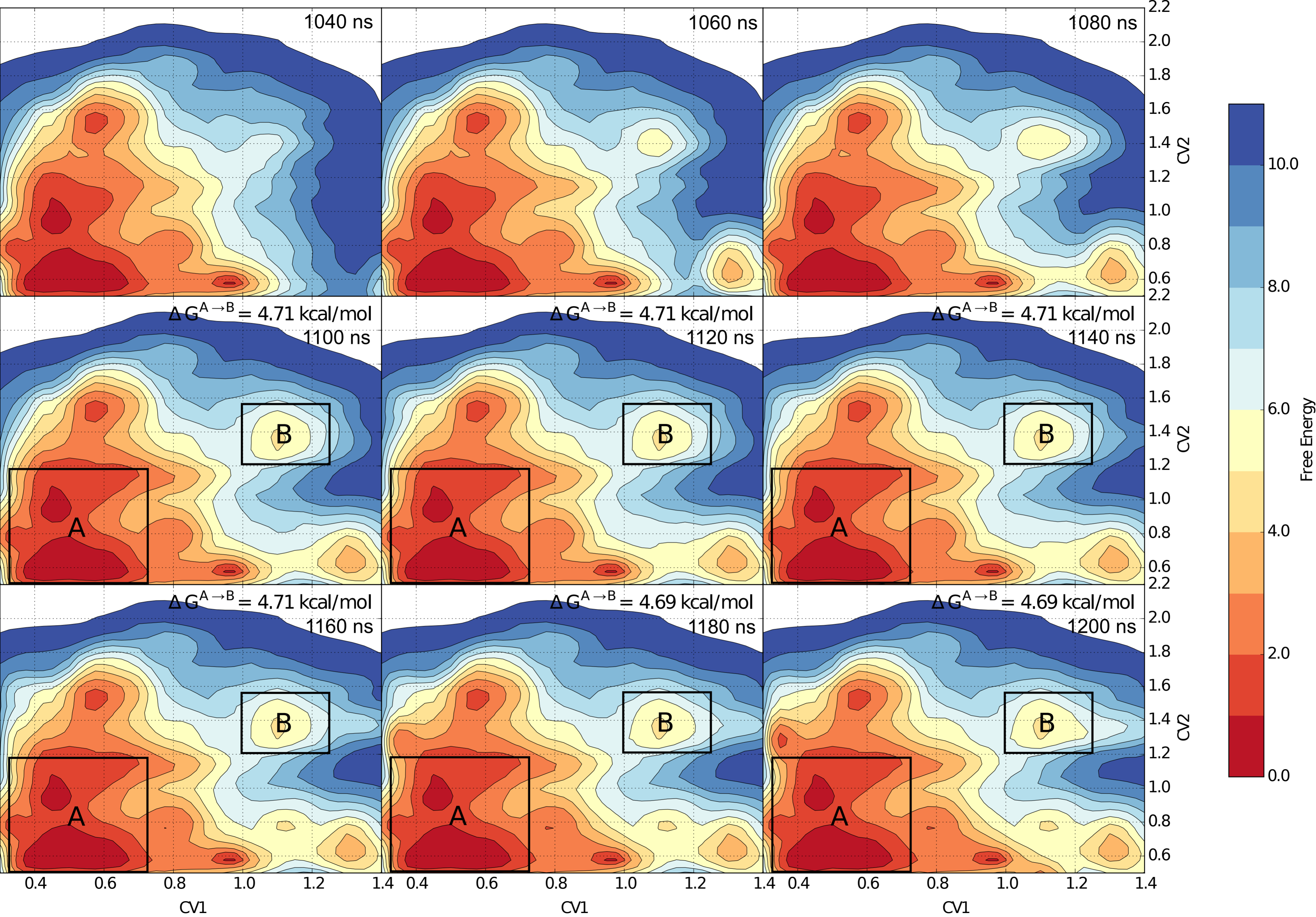

Supplement: S15 Fig — Bidimensional profiles of T315I along CV1 and CV2 calculated at intervals of 20 ns in the last 160 ns of simulation time. (PDF) [file pcbi.1004578.s015.pdf]

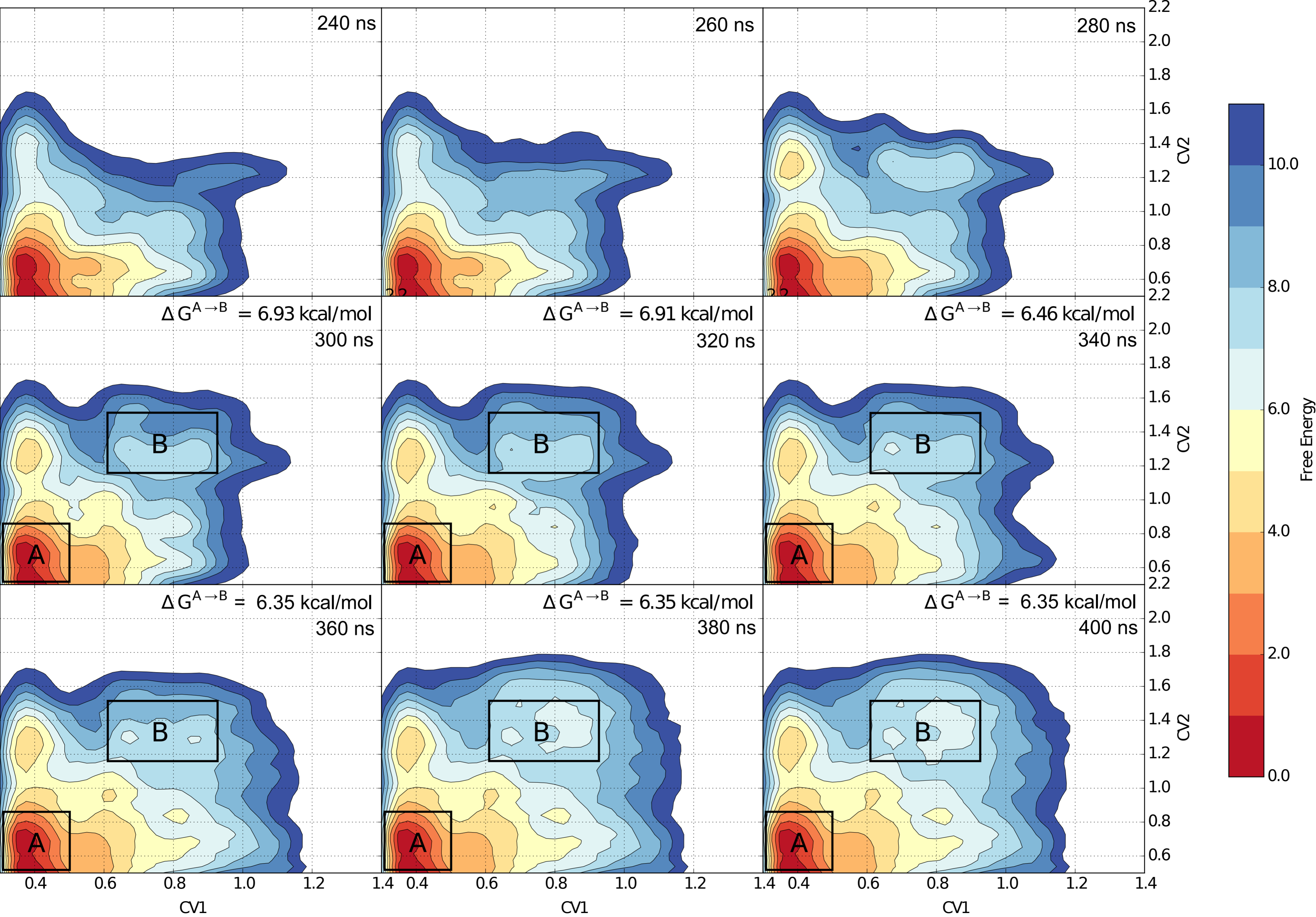

Supplement: S16 Fig — Bidimensional profiles of E450K along CV1 and CV2 calculated at intervals of 20 ns in the last 160 ns of simulation time. (PDF) [file pcbi.1004578.s016.pdf]

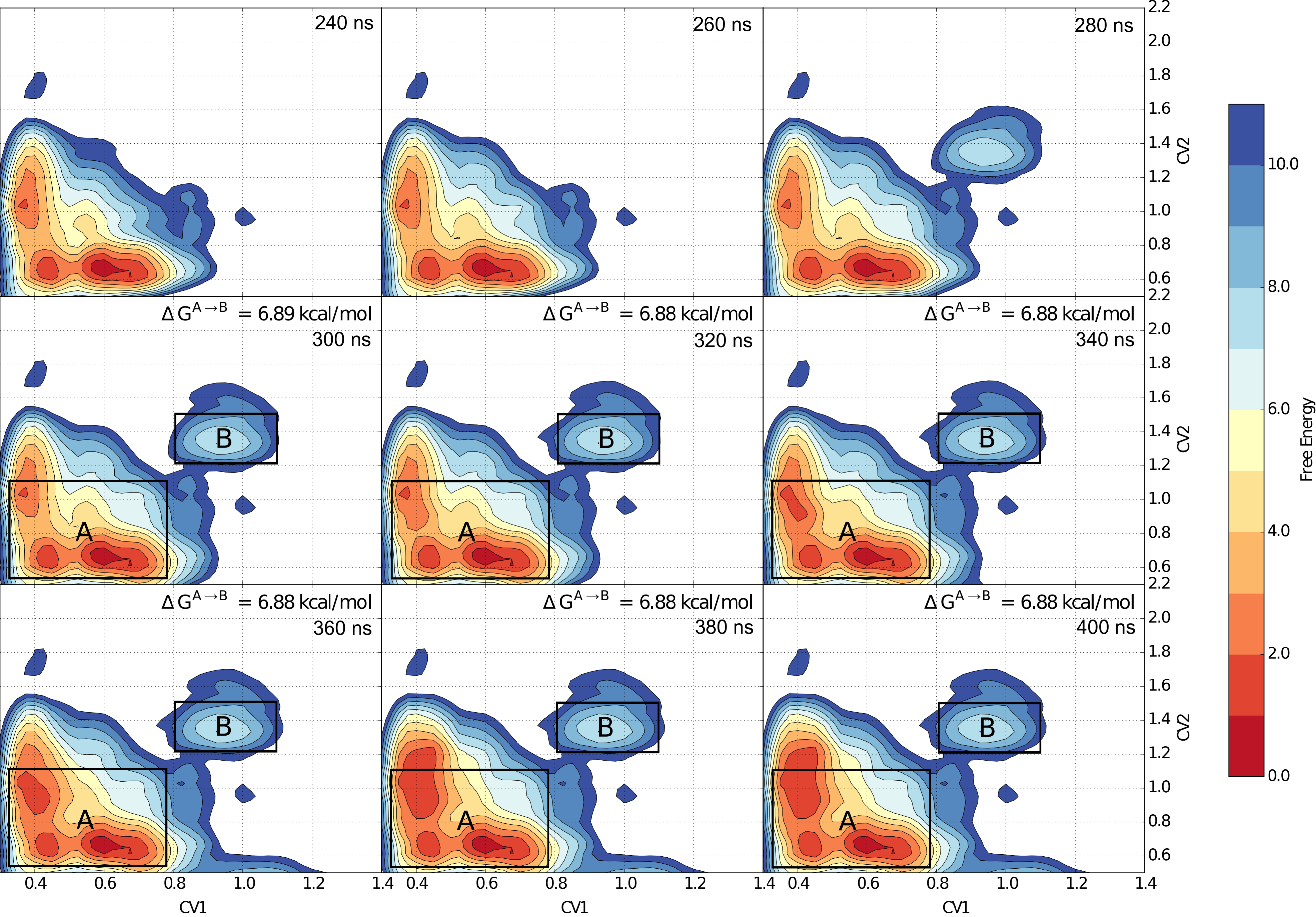

Supplement: S17 Fig — Bidimensional profiles of H396P along CV1 and CV2 calculated at intervals of 20 ns in the last 160 ns of simulation time. (PDF) [file pcbi.1004578.s017.pdf]

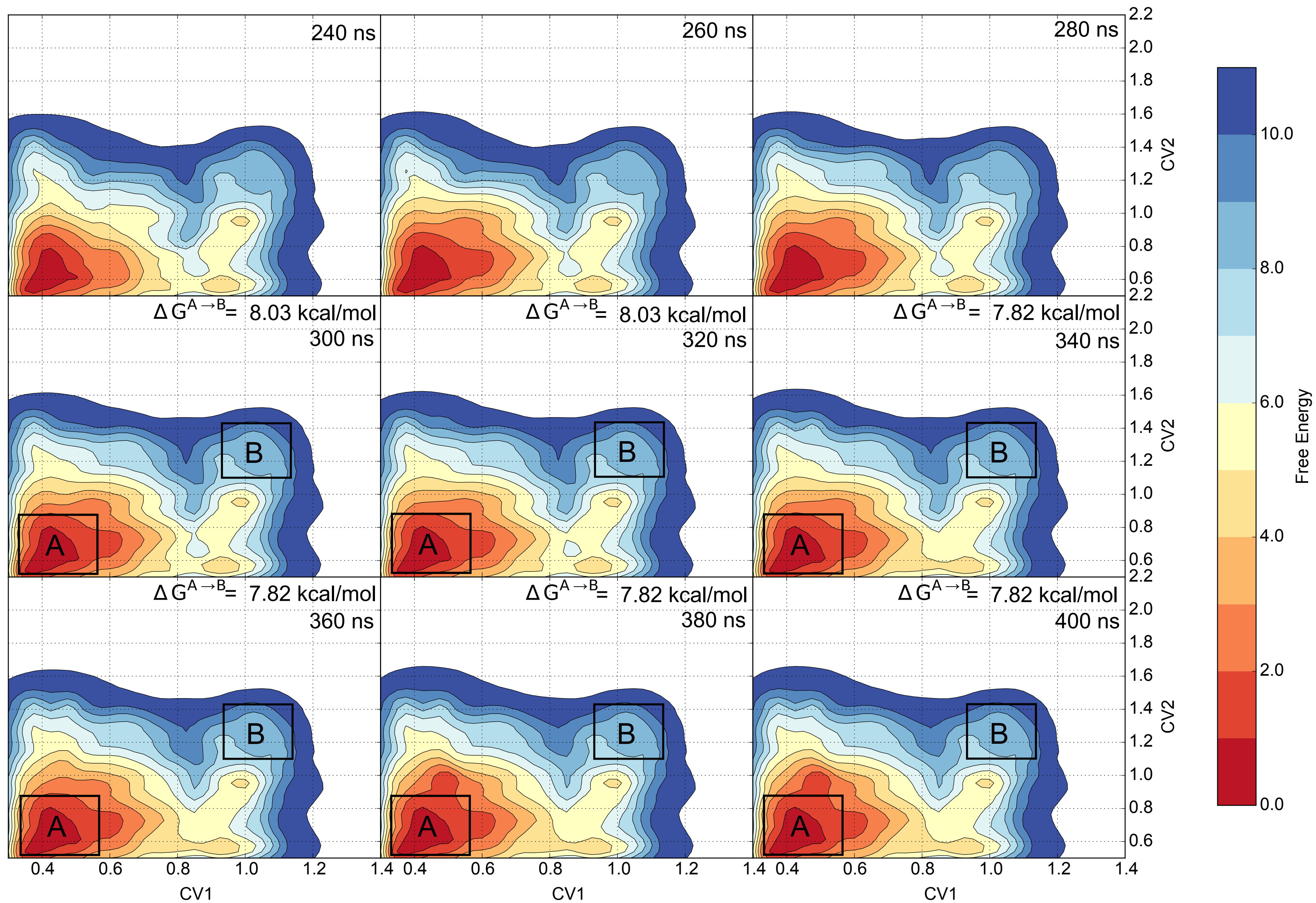

Supplement: S18 Fig — Bidimensional profiles of E279K along CV1 and CV2 calculated at intervals of 20 ns in the last 160 ns of simulation time. (PDF) [file pcbi.1004578.s018.pdf]
